# Three‐Week Video‐ and Home‐Based Training Program for People with Ataxia: A Pilot Randomized Controlled Trial

**DOI:** 10.1002/mdc3.70225

**Published:** 2025-07-14

**Authors:** Clara Rentz, Alisha Reinhardt, Naomi Jung, Vignesh Vanchinathan, Jutta Peterburs, Heike Jacobi, Dagmar Timmann, Andreas Thieme, Doris Brötz, Christian Bellebaum, Alfons Schnitzler, Katrin Amunts, Martina Minnerop

**Affiliations:** ^1^ Institute of Neuroscience and Medicine (INM‐1), Research Centre Jülich Jülich Germany; ^2^ Whiting School of Engineering John Hopkins University Baltimore Maryland USA; ^3^ Institute of Systems Medicine and Department of Human Medicine, MSH Medical School Hamburg Hamburg Germany; ^4^ Department of Neurology University Hospital of Heidelberg Heidelberg Germany; ^5^ Department of Neurology and Center for Translational Neuro‐ and Behavioral Sciences (C‐TNBS), University Hospital Essen University of Duisburg‐Essen Duisburg Germany; ^6^ Institute of Medical Psychology and Behavioral Neurobiology University of Tübingen Tübingen Germany; ^7^ Faculty of Mathematics and Natural Sciences Heinrich Heine University Düsseldorf Düsseldorf Germany; ^8^ Department of Neurology, Center for Movement Disorders and Neuromodulation, Medical Faculty Heinrich Heine University Düsseldorf Düsseldorf Germany; ^9^ Institute of Clinical Neuroscience and Medical Psychology, Medical Faculty & University Hospital Düsseldorf Heinrich Heine University Düsseldorf Düsseldorf Germany; ^10^ C. and O. Vogt Institute for Brain Research, Medical Faculty, University Hospital Düsseldorf Heinrich‐Heine‐University Düsseldorf Germany

**Keywords:** ataxia, training, rehabilitation, video‐based, home‐based, RCT

## Abstract

**Background:**

Gait and balance impairment is a disabling clinical feature in people with degenerative cerebellar ataxia.

**Objectives:**

We performed a rater‐blinded, parallel 3‐arm randomized controlled trial with delayed‐start‐design (exploratory proof‐of‐concept study) to assess whether, compared to control, people with mild/moderate hereditary ataxia benefit from additional video‐based training with different frequencies, and how clinical characteristics interact with training success.

**Methods:**

Digital gait/balance measures were assessed before and after a three‐week video‐based training program at home (Train20: 4 × 20min/week, *n* = 11; Train40: 2 × 40min/week, *n* = 11; control: standard medical care, *n* = 10). Group differences at baseline and changes over time were assessed using ANOVA. Linear mixed models were conducted to examine the influence of clinical variables on outcomes over time. Further exploratory analyses were performed using intraclass correlation coefficients (ICC), and paired t‐tests within each group.

**Results:**

All variables showed good to excellent test–retest reliability (ICC ≥0.69). No significant interactions between group and measurement time were found for clinical or gait/balance variables (*p* ≥ 0.338). However, participants with higher initial disease severity, greater impairment in activities of daily living, and better well‐being showed significant improvements in feet‐together stance (*p* = 0.04), normal (*p* = 0.01), and backward gait (*p* = 0.03). Exploratory analyses showed improvement only in Train40.

**Conclusions:**

Although the protocol did not lead to general improvements in people with mild/moderate ataxia, irrespective of training frequency, those with higher initial disease severity, higher functional impairment, and better mental well‐being showed significant benefits. Greater attention should be given to the impact of well‐being to enhance motor training outcomes. Longer, less frequent sessions may offer greater potential for improvement.

Progressive impairments in gait and balance are the main challenges for people with hereditary cerebellar ataxia in everyday life, resulting in reduced mobility, increased risk of falls and fall‐induced injuries, decreased independence, and a substantial impact on quality of life.^1^, ^2^ According to reports from those affected, exercise and physiotherapy are among the most effective supportive measures that are currently available.^3^ Targeted and intense training programs lasting one to 6 months, including gait and balance training, have shown potential to improve functional mobility, reduce fall risk, and improve well‐being.^4^, ^5^ However, these in‐person approaches are only suitable and accessible for a subset of participants, necessitating effective training and monitoring solutions that require little or no physical presence at the study center.^6^, ^7^ Shorter or home‐based training regimens, eg, 4 weeks of coordinative training, led to short‐ and long‐term improvements in clinical disease severity scores and quantitative gait and balance parameters in people with cerebellar ataxia.^8^, ^9^ Insights into remote training interventions are limited. A systematic review on rehabilitation programs in people with genetic degenerative ataxia^10^ summarized that only four out of 17 studies included home‐based training, one of which was a randomized controlled trial (RCT). Apart from one recent publication (*n* = 76),^11^ other training studies had relatively low sample sizes (*n* = 8–17).^12^, ^13^, ^14^, ^15^ Most of the studies used clinical scores, while quantitative gait and balance parameters are considered as more sensitive methods to depict functional improvements.^16^, ^17^

Defining training parameters (frequency, duration, intensity) is crucial for optimizing rehabilitation outcomes.^11^ As yet, no direct comparison of training frequency has been reported in people with cerebellar ataxia. Standardized guidelines are lacking, and further RCTs are needed to optimize home‐based training programs for hereditary cerebellar ataxia.

Here, we present an exploratory proof‐of‐concept RCT with comparably high sample size and the primary aim of exploring the impact of home‐based, video‐based training on gait and balance in people with hereditary degenerative cerebellar ataxia, compared to a control group receiving standard medical care without additional training. We further analyzed test–retest‐reliability; whether the distribution of training sessions across the week, with consistent weekly training time and content, affects the training effect; and how clinical characteristics interact with training success.

We hypothesized that video‐based training at home would result in significant improvements in gait and balance abilities. In addition, we hypothesized that shorter, more frequent training sessions would lead to greater improvements compared to longer, less frequent ones, and that clinical characteristics, such as disease severity and baseline functional status, would be key determinants of individual training success.

## Methods

### Participants

Participants with confirmed hereditary ataxia—indicated by genetic diagnosis, positive family history, or early‐onset symptoms—were recruited at the University Hospitals Düsseldorf, Essen, and Heidelberg, via the German Heredo‐Ataxia Society (Deutsche Heredo‐Ataxie‐Gesellschaft e.V.), and via social media. Subjects unable to walk unassisted for at least 2 min or with secondary central nervous system diseases were excluded. Written informed consent was obtained in agreement with the Declaration of Helsinki, and the study protocol was approved by the ethics committee of the Medical Faculty of the Heinrich Heine University Düsseldorf, Germany (2022–1994). The study was preregistered at ClinicalTrials.gov (NCT06617884).

### Study Design/Interventions

**Figure 1:**
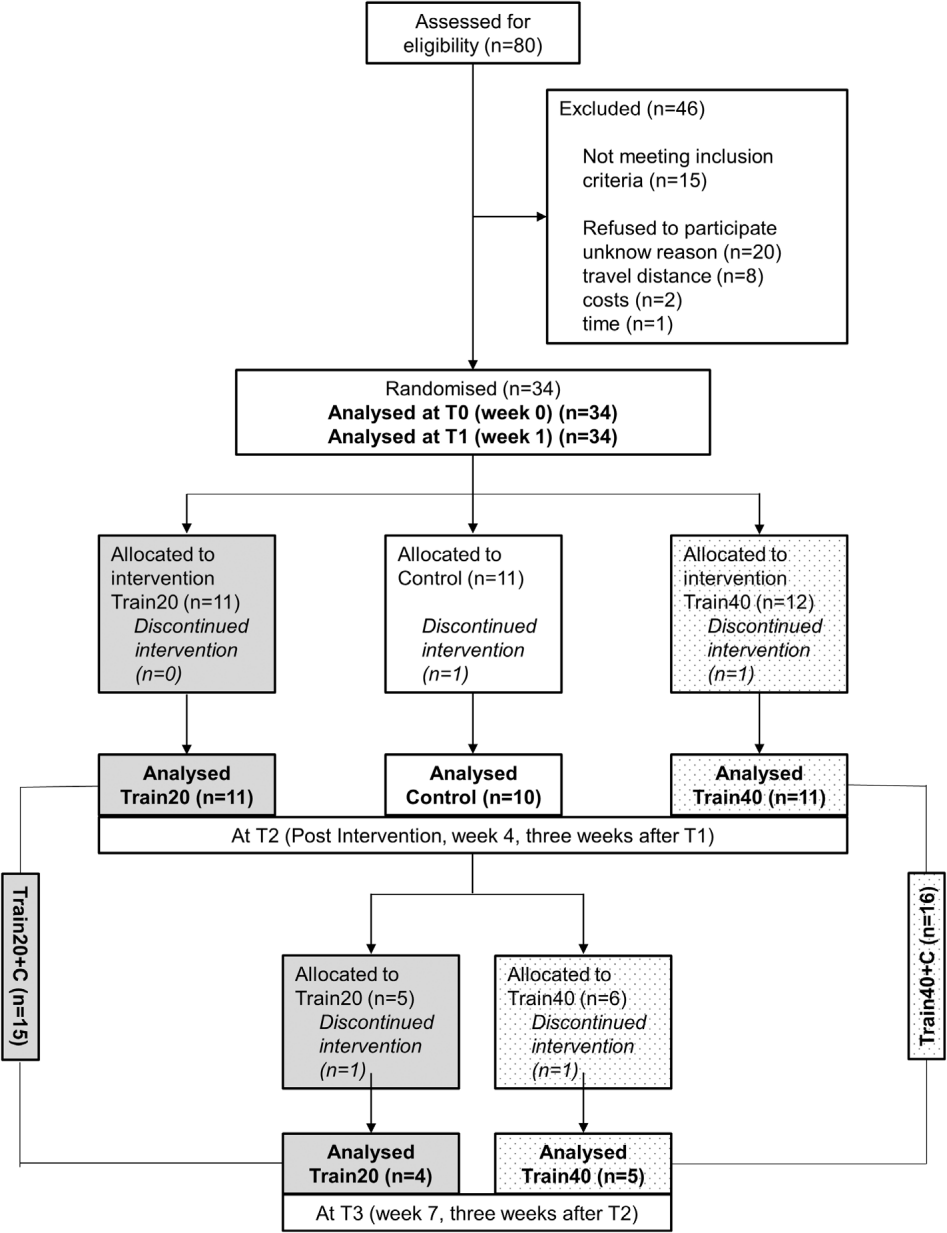
Flow of participants through the trial (CONSORT flow diagram), including group assignments for analysis.

The study comprised three mandatory visits: T0 (screening), T1 (baseline), and T2 (post‐training, see Fig. 1). All participants took part in a one‐week baseline phase without training (T0 to T1) for familiarization^18^ and test–retest reliability assessment. They were then randomly assigned to one of two training protocols or a control group (Train20, Train40, Control) in a 1:1:1 ratio using a computer‐generated random allocation sequence (R software, version 4.3.1). Group assignment was not concealed from the enrolling investigator; however, the examiner performing the clinical assessments remained blinded to group allocation. After T2, participants in the control group also underwent the training and completed a fourth study visit (T3, after week 7). To increase statistical power, data from the control group was subsequently merged with their respective training groups from the first phase, forming two extended groups: Train20 combined with former control participants (Train20 + C) and Train40 combined with former control participants (Train40 + C).

### Training

In the 3 weeks between baseline and post‐training, participants followed uniform, pre‐recorded training videos at home, watching them on a computer or laptop and performing the exercises simultaneously. The videos were designed to complement (and not to replace) individual training or physiotherapy. Exercises, designed with an ataxia physiotherapy expert (D.B.), included easy‐to‐perform coordination, strength, and mobility exercises in each video/training session (see Supplementary Material), and were designed to minimize fall risk in the unsupervised home setting. Both training protocols had identical content and total weekly duration but differed in session frequency and length: Train20 performed four 20‐minute sessions per week, while Train40 completed two 40‐minute sessions, by combining two 20‐minute video segments (eg, segments 1 + 2 and 3 + 4) per session to streamline the program. The total training duration and session length were in alignment with standard rehabilitation measures in Germany, typically lasting 3 weeks (inpatient setting) or 6–12 sessions of approximately 20 minutes (outpatient setting). This was supported by literature suggesting that study compliance tends to decline after 3 weeks in healthy older adults,^19^ and that supervised short‐term training interventions lasting 5 days to 4 weeks have already shown benefits in people with cerebellar ataxia.^8^, ^9^, ^20^ All participants, including the control group without additional training, maintained their standard medical care routine, including physiotherapy, prescribed medications, and other exercises.

### Assessments

Following current guidance documents^21^ and consensus recommendations,^22^, ^23^ we combined clinician‐reported outcomes (ClinRO) with patient‐reported outcomes (PRO), and quantitative performance outcomes (PerfO).

#### Baseline demographics and variables (T0)

Medical history, handedness, physical/sport activity (amount per week/day), amount of physiotherapy, occupational therapy and speech therapy per week, estimated number of (almost) falls (past 6 months and 1 week), and years of education (school plus further education) were collected via questionnaire. Depressive and anxiety symptoms were surveyed with the depression module of the Patient Health Questionnaire (PHQ‐9^24^). We assessed cognitive impairment (Montreal Cognitive Assessment^25^, ^26^), and presence or absence of non‐ataxia symptoms (INAS‐score, inventory of non‐ataxia signs^27^).

#### Longitudinal ClinRO and PRO (T0–T3)

For ClinROs, we assessed the severity of ataxia (SARA‐score, scale for the assessment and rating of ataxia^28^), functional mobility (Timed Up and Go test, TUG^29^), and impairment of daily life activities (activities of daily living section of the Friedreich's ataxia rating scale, FARS‐ADL^30^). The PROs included questionnaires on habitual well‐being (FAHW^31^), and activities‐specific balance confidence (ABC‐D^32^) (details in Supplementary Material).

#### Longitudinal PerfO (T0–T3)

Three gait tasks and five balance tasks (bipedal and unipedal stance conditions) were performed at each study visit to assess gait and balance: Normal gait (NG), backward gait (BG), tandem gait (TG), natural stance (NS), feet‐together stance (FTS), tandem stance (TS), FTS with eyes closed (FTSec), and single‐leg stance (SS), recorded with a force plate (zebris Medical GmbH, Isny, Germany).^33^ NG was conducted for 2 minutes, BG and TG for four lanes (each 4.24 m), and each balance task was attempted for 30 seconds without support, with values under 30 seconds excluded from longitudinal analyses. We only analyzed tasks completed by at least 75% of participants^34^ (≥30s for balance tasks, gait without support). Outcomes to quantify gait performance included stride time [s], double support proportion [%], mean velocity [m/s], external rotation of the feet [°], step width [cm], and step width SD [cm]. Balance outcomes included sway area [mm^2^] and sway velocity [mm/s] (for details see Supplementary Material). The primary outcome was NG velocity.

After the training, perceived change in gait and balance was retrieved via a Patient Global Impression of Change (PGIC) questionnaire.

### Statistical Methods

Based on reported effect size and SD in gait velocity,^4^ we estimated a sample size of 17 participants per group to achieve 80% power for detecting pre‐ to post‐training changes. Since this sample size was not reached, this study is considered a pilot RCT.

Statistical analyses were performed using R software (version 4.3.1) and IBM SPSS Statistics (version 27). We report uncorrected (*p*‐unc, values ≤0.05 considered significant), and Bonferroni‐corrected *p*‐values for multiple comparisons (*p*‐corr, values *≤*0.05/n, where n is the number of comparisons, considered significant). Missing values were excluded, while outliers were retained, as they reflect the natural variability in this population.

*Reliability (whole sample)*. For all PerfO, intraclass‐correlation coefficients (ICCs) with two‐way random effects models were calculated to confirm stability and reliability of the gait and balance measures (PerfO) between T0 and T1 (screening and baseline visit), ensuring that observed changes over time are due to true score differences rather than random error. The minimal detectable change (MDC) of PerfO was calculated between T0 and T1 (MDC = 1.96 * SEM * √2) at the 95% confidence level (SEM = standard error of measurement) and changes within the whole group in PerfO between T0 and T1 were identified using dependent samples t‐tests (*p*‐corr*≤*0.05/20).

*Baseline comparisons*. For comparison of baseline demographics/variables, and PRO and ClinRO between groups, we used a univariate ANOVA (three groups) or independent t‐tests (two groups; *p*‐corr*≤*0.05/17). Categorical variables were analyzed with chi‐square tests.

*Analysis of the training effect*. To investigate group differences in PerfO, ClinRO, and PRO across the intervention phase, a series of 2 × 3 ANOVA with within‐subjects factor “time point” (pre, post), and between‐subjects factor “group” (Train20, Train40, Control), was performed (primary analysis). Each dependent variable (*n* = 18 for PerfO; *n* = 5 for ClinRO/PRO, *p*‐corr*≤*0.05/18 or *p*‐corr*≤*0.05/5), was tested for main effects of group, time point, and their interaction. Similarly, a series of 2 × 2 ANOVA was conducted for comparisons between the extended training groups (Train20 + C, Train40 + C) across time. An exploratory (secondary) analysis compared pre‐ and post‐training values within each group using dependent samples t‐tests. For the primary outcome gait velocity, independent contrasts of improvement were calculated between the groups (independent t‐tests) and are reported in the Supplementary Material.

*Interaction of ClinRO/PRO and PerfO*. For all participants who had completed training (Train20 + C plus Train40 + C, *n* = 31), a linear mixed model was used to assess whether baseline values of ClinROs and PROs influenced changes in PerfO across the intervention phase (primary analysis, the sample size of *n* = 31 does not apply to FTS/FTSec, as not all participants completed this task). The model included fixed effects for ClinRO/PRO, time point (T1, T2), and their interaction to examine their influence on change of performance outcomes over time. To account for the repeated measures design, a random intercept for individual subjects was included, capturing individual variability in baseline levels. This model was applied separately for all PerfOs (*p*‐corr*≤*0.05/18). For those models that revealed a significant interaction effect, further analyses were conducted to resolve the interactions. Hence, estimated marginal means for gait or balance at each time point were calculated, specifically examining the effects at higher and lower levels of the clinical scores (one SD above and below the mean). Pairwise contrasts were conducted to further explore the differences between the estimated marginal means at T1 and T2 (*p*‐corr*≤*0.05/2).

## Results

Of 80 people with hereditary cerebellar ataxia assessed for eligibility, 34 completed the first two study visits, and 31/34 completed the training (see Fig. 1 for dropouts). All participants had mild to moderate cerebellar ataxia (SARA‐score 8.4 ± 3.5) and a mean disease duration of 9.2 ± 8.3 years (Table 1, Table S1). The frequency of impaired vibration sense is reported in Table S2. TS, SS, and TG had completion rates of <75% and were not evaluated.

**TABLE 1:** Comparison of demographic and clinical characteristics between the groups at baseline

	Whole group	Train20	Train40	Control	ANOVA
Sample size	*n* = 34	*n* = 11	*n* = 11	*n* = 10	
Sex (*n* female)	16	6	6	2	
Handedness (*n* left‐handed)	1	0	1	0	
Number of pure cerebellar ataxias	21	7	5	9	
Age‐(years)	54.8 ± 11.5 (27–73)	52.8 ± 12.7 (32–66)	56.6 ± 8.6 (40–67)	57.9 ± 12.8 (27–73)	*p* = 0.573
Height (cm)	173.1 ± 8.1 (157–190)	172.3 ± 9.0 (157–185)	174.6 ± 6.9 (163–187)	173.4 ± 9.5 (162–190)	*p* = 0.822
Weight (kg)	73.1 ± 14.5 (50–108) *n* = 29	69.3 ± 16.3 (50–94) *n* = 10	69.8 ± 8.3 (58–83) *n* = 9	82.4 ± 14.5 (65–108) *n* = 9	*p* = 0.083
Disease duration (years)	9.2 ± 8.3 (0–36) *n* = 32	10.1 ± 8.2 (0–25) *n* = 11	7.0 ± 6.2 (1–21) *n* = 9	11.7 ± 10.7 (1–36) *n* = 10	*p* = 0.441
Age at disease onset (years)	45.6 ± 14.4 (12–68)	42.7 ± 14.5 (18–60)	49.6 ± 11.1 (34–65)	46.2 ± 18.5 (12–68)	*p* = 0.560
Education (years)	15.5 ± 2.9 (11–23)	13.9 ± 2.0 (11–18)	16.9 ± 2.8 (12–22)	15.7 ± 3.5 (13–23)	*p* = 0.055
PHQ‐9 score (/27)	6.8 ± 4.0 (1–19)	6.5 ± 3.2 (2–11)	4.9 ± 3.6 (1–12)	9.0 ± 4.4 (4–19)	*p* = 0.057
MOCA score (/30)	25.0 ± 3.9 (11–30)	25.0 ± 3.1 (19–30)	26.0 ± 2.4 (21–29)	25.6 ± 3.8 (18–29)	*p* = 0.753
Sport per week (days)	4.2 ± 2.2 (0–7)	4.2 ± 1.6 (2–7)	4.0 ± 2.6 (0–7)	4.4 ± 2.8 (0–7)	*p* = 0.926
Sport per day (hours)	1.6 ± 1.2 (0–6 *n* = 33	2.3 ± 1.6 (1–6) *n* = 11	1.2 ± 0.7 (0–2.5) *n* = 11	1.3 ± 1.1 (0–3) *n* = 9	*p* = 0.064
Physiotherapy per week (amount)	1.4 ± 0.8 (0–3)	1.6 ± 0.8 (0–3)	1.1 ± 0.8 (0–2	1.4 ± 0.8 (0–2)	*p* = 0.435
Occupational therapy per week (amount)	0.4 ± 0.6 (0–2)	0.5 ± 0.7 (0–2)	0.2 ± 0.4 (0–1)	0.4 ± 0.7 (0–2)	*p* = 0.459
Speech therapy per week (amount)	0.4 ± 0.8 (0–3)	0.5 ± 0.9 (0–3)	0.2 ± 0.4 (0–1)	0.2 ± 0.4 (0–1)	*p* = 0.447
Falls within 6 months (amount)	3.4 ± 7.8 (0–40) *n* = 30	3.2 ± 5.4 (0–18) *n* = 10	1.8 ± 2.2 (0–6) *n* = 11	6.7 ± 14.8 (0–40) *n* = 7	*p* = 0.461
Almost falls within 6 months (amount)	19.0 ± 66. (0–365) *n* = 30	44.2 ± 113.5 (0–365) *n* = 10	7.3 ± 8.5 (0–25) *n* = 11	6.9 ± 8.9 (0–24) *n* = 7	*p* = 0.402
Falls within 1 week (amount)	0.5 ± 1.9 (0–10) *n* = 31	0.4 ± 0.9 (0–3) *n* = 11	0.1 ± 0.3 (0–1) *n* = 10	1.3 ± 3.5 (0–10) *n* = 8	*p* = 0.441
Almost falls within 1 week (amount)	1.9 ± 5.0 (0–20) *n* = 31	2.3 ± 5.6 (0–18) *n* = 11	2.0 ± 6.3 (0–20) *n* = 10	1.6 ± 2. (0–8) *n* = 8	*p* = 0.966

*Note*: ANOVA – univariate ANOVA between the three smaller groups, Pure cerebellar ataxias = hereditary ataxias commonly regarded as “pure” cerebellar ataxias (eg, SCA6, SCA14 etc.); Disease duration = Years since disease onset; Education = Educational level measured in years of Education; *n* = available sample size for the respective variable if not available for the entire group; Sport per week/day = Amount of physical/sport activity perceived as demanding, per week or per day; PHQ‐9 = depression module of the Patient Health Questionnaire; MOCA = cognitive impairment according to the Montreal Cognitive Assessment Test.

*Reliability (whole sample)*. Most variables demonstrated good to excellent reliability (ICCs 0.69–0.96, and 0.94 for primary outcome NG velocity), indicating consistent measurement across the two time points. No significant difference was found between T0 and T1 according to pairwise t‐tests (all *p* ≥ 0.051). ICC and MDC values are reported in Table S3.

*Baseline comparisons*. Train20, Train40 and control group did not differ significantly in any of the baseline variables (univariate ANOVA, all *p* ≥ 0.055, see Table 1) or in ClinRO and PRO scores at baseline (*p* ≥ 0.139). Within the extended training groups, a higher balance confidence (t‐test, *p*‐unc = 0.050), and a higher educational level (*p*‐unc = 0.004) were observed in Train40 + C compared to Train20 + C (see Tables S4 and S5).

*Analysis of the training effect (primary analysis)*. Mean descriptive values for dependent variables assessed longitudinally (ClinRO, PRO, and PerfO, pre‐ and post‐training) can be found in Table 2. For each variable, separate ANOVA were conducted to determine whether there were changes over time (before and after training), dependent on groups (Train20, Train40, control). No significant interaction effect of “time × group” was found for the primary outcome NG velocity (*p* = 0.880) or for any other of the dependent variables (all *p* ≥ 0.602, see Table 2). More detailed analyses for the primary outcome gait velocity are reported in the Supplementary Material. Significant main effects of group indicated generally lower balance confidence in Train20 compared to Train40 (*p*‐unc = 0.043) and to control group (*p*‐corr = 0.008), and a generally lower step width in NG in the control group compared to Train20 (*p*‐unc = 0.029). No significant main effects of time were found (all *p* ≥ 0.244). In the PGIC (*n* = 30, range − 3 to 3), 40% of all participants who underwent training (Train20 + C, Train40 + C) reported a 1 to 2‐point improvement in both gait and balance, 56.7% reported no change, and 3.3% (one participant) reported a 1‐point worsening.

**TABLE 2:** Comparison of longitudinal ClinRO, PRO and PerfO gait and balance variables between the groups before and after training

Variable	Mean ± SD (Min.–Max.)			Interaction Effect (G*T), Main Effect Group (G), Main Effect Time (T)
	Control, *n* = 10	Train20, *n* = 11	Train40, *n* = 11	
SARA score pre	8.2 ± 3.3	9.6 ± 3.5	7.9 ± 3.8	G*T: *p* = 0.976 G*: p* = 0.170 T*: p* = 0.434
	(5–16)	(3–15)	(2.5–15)	
SARA score post	7.5 ± 5.1	9.1 ± 2.4	6.9 ± 2.8	
	(0.5–17.5)	(5.5–13.5)	(2.5–11.5)	
ADL score pre	7.5 ± 5.3	8.9 ± 3.8	8.5 ± 3.8	G*T: *p* = 0.999 G*: p* = 0.601 T*: p* = 0.460
	(1–15)	(2–13)	(3–14)	
ADL score post	6.8 ± 4.7	8.0 ± 4.3	7.7 ± 3.6	
	(0–15)	(1–14)	(4–15)	
TUG time pre	11.4 ± 3.7 s	13.1 ± 5.7 s	10.7 ± 3.4 s	G*T: *p* = 0.835 G*: p* = 0.063 T*: p* = 0.956
	(7.5–18.2)	(6.2–26.5)	(4.7–15.2)	
TUG time post	11.2 ± 4.3 s	14.0 ± 5.2 s	10.1 ± 3.4 s	
	(5.9–17.5)	(7.9–24.3)	(4.5–17.1)	
ABC‐D score pre	68.63 ± 16.07	54.29 ± 15.04	67.68 ± 18.51	G*T: *p* = 0.982 G*: p* = 0.007** T*: p* = 0.803
	(45.0–89.1)	(31.6–76.3)	(30.0–86.3)	
ABC‐D score post	67.31 ± 13.24	52.71 ± 10.99	64.72 ± 15.34	
	(49.0–81.0)	(34.0–66.0)	(36.0–86.0)	
FAHW score pre	28.40 ± 20.23	29.91 ± 25.28	32.45 ± 18.79	G*T: *p* = 0.910 G*: p* = 0.623 T*: p* = 0.855
	(−5–61)	(−11–58)	(5–59)	
FAHW score post^a^	25.90 ± 22.39	26.60 ± 30.61	34.82 ± 19.78	
	(2–73)	(−15–73)	(5–64)	
NG stride time pre	1.22 ± 0.10 s	1.18 ± 0.15 s	**1.18 ± 0.11 s**	G*T: *p* = 0.960 G*: p* = 0.457 T*: p* = 0.455
	(1.09–1.37)	(0.95–1.51)	**(1.01–1.30)**	
NG stride time post	1.20 ± 0.12 s	1.17 ± 0.13 s	**1.14 ± 0.11 s**	
	(1.06–1.41)	(0.92–1.40)	**(0.99–1.32)**	
NG double supp. pre	34.34 ± 6.59%	35.61 ± 6.12%	**32.98 ± 2.48%**	G*T: *p* = 0.942 G*: p* = 0.209 T*: p* = 0.726
	(26.8–48.4)	(26.8–49.1)	**(29.5–36.9)**	
NG double supp. post	33.77 ± 7.48%	35.74 ± 8.43%	**31.84 ± 2.76%**	
	(27.4–52.3)	(26.1–57.0)	**(27.6–36.0)**	
NG velocity pre	0.79 ± 0.14 m/s	0.74 ± 0.20 m/s	**0.83 ± 0.15 m/s**	G*T: *p* = 0.880 G*: p* = 0.079 T*: p* = 0.411
	(0.53–1.00)	(0.39–1.08)	**(0.50–0.97)**	
NG velocity post	0.83 ± 0.18 m/s	0.75 ± 0.22 m/s	**0.89 ± 0.17 m/s**	
	(0.47–1.03)	(0.36–1.11)	**(0.56–1.08)**	
NG foot rot. pre	15.6 ± 6.5 °	11.3 ± 7.1 °	11.5 ± 4.0 °	G*T: *p* = 0.990 G*: p* = 0.055 T*: p* = 0.932
	(5.1–25.5)	(3.2–23.6)	(4.0–18.0)	
NG foot rot. post	15.5 ± 6.3 °	11.70 ± 7.4 °	11.6 ± 4.3 °	
	(4.6–24.1)	(2.3–24.9)	(5.6–18.4)	
NG step width pre	**15.80 ± 2.70 cm**	17.82 ± 3.82 cm	17.36 ± 4.39 cm	G*T: *p* = 0.602 G*: p* = 0.038* T*: p* = 0.631
	**(12–21)**	(11–23)	(12–26)	
NG step width post	**15.10 ± 2.18 cm**	18.55 ± 2.98 cm	16.09 ± 3.51 cm	
	**(13–20)**	(13–23)	(12–25)	
NG step width SD pre	3.70 ± 1.25 cm	3.45 ± 1.37 cm	4.09 ± 1.04 cm	G*T: *p* = 0.737 G*: p* = 0.290 T*: p* = 0.918
	(2–6)	(2–7)	(3–6)	
NG step width SD post	4.00 ± 1.05 cm	3.36 ± 1.12 cm	3.82 ± 1.33 cm	
	(3–6)	(2–6)	(2–6)	
BG stride time pre	1.42 ± 0.36 s	1.42 ± 0.14 s	1.33 ± 0.21 s	G*T: *p* = 0.918 G*: p* = 0.450 T*: p* = 0.388
	(1.14–2.39)	(1.22–1.64)	(0.94–1.77)	
BG stride time post	1.33 ± 0.23 s	1.38 ± 0.17 s	1.30 ± 0.20 s	
	(1.10–1.80)	(1.17–1.71)	(0.96–1.60)	
BG double supp. pre	51.91 ± 11.29%	49.93 ± 10.99%	44.49 ± 7.73%	G*T: *p* = 0.911 G*: p* = 0.085 T*: p* = 0.704
	(36.6–68.2)	(38.0–74.8)	(30.3–52.7)	
BG double supp. post	49.40 ± 10.94%	49.75 ± 10.92%	44.25 ± 6.39%	
	(35.8–69.9)	(39.5–75.6)	(32.5–51.9)	
BG velocity pre	0.39 ± 0.12 m/s	0.33 ± 0.14 m/s	0.40 ± 0.15 m/s	G*T: *p* = 0.962 G*: p* = 0.173 T*: p* = 0.451
	(0.25–0.64)	(0.11–0.50)	(0.19–0.64)	
BG velocity post	0.44 ± 0.15 m/s	0.35 ± 0.15 m/s	0.42 ± 0.15 m/s	
	(0.19–0.69)	(0.11–0.56)	(0.22–0.75)	
BG foot rot. pre	6.0 ± 6.5 °	3.9 ± 4.3 °	5.0 ± 6.0 °	G*T: *p* = 0.991 G*: p* = 0.480 T*: p* = 0.985
	(−1.4–17.4)	(−3.1–11.4)	(−5.5–14.8)	
BG foot rot. post	5.8 ± 4.3 °	4.0 ± 4.6 °	5.2 ± 5.4 °	
	(−1.2–12.7)	(−3.9–10.2)	(−4.1–14.2)	
BG step width pre	25.30 ± 5.14 cm	24.90 ± 4.25 cm	**25.55 ± 4.34 cm**	G*T: *p* = 0.993 G*: p* = 0.927 T*: p* = 0.604
	(15–33)	(18–30)	**(19–35)**	
BG step width post	24.60 ± 3.69 cm	24.50 ± 4.45 cm	**24.91 ± 4.28 cm**	
	(17–31)	(18–31)	**(18–34)**	
BG step width SD pre	3.40 ± 1.58 cm	2.70 ± 0.67 cm	2.82 ± 1.47 cm	G*T: *p* = 0.926 G*: p* = 0.163 T*: p* = 0.619
	(2–7)	(2–4)	(1–6)	
BG step width SD post	3.60 ± 1.51 cm	3.00 ± 1.05 cm	2.82 ± 1.08 cm	
	(2–6)	(2–5)	(1–5)	
NS sway area pre	1212 ± 1409 mm^2^	820 ± 696 mm^2^	837 ± 911 mm^2^	G*T: *p* = 0.753 G*: p* = 0.463 T*: p* = 0.244
	(77–4045)	(208–2394)	(190–3036)	
NS sway area post	797 ± 804 mm^2^	788 ± 805 mm^2^	495 ± 393 mm^2^	
	(86–2854)	(252–3093)	(134–1275)	
NS sway velocity pre	35.6 ± 45.4 mm/s	22.8 ± 13.1 mm/s	20.6 ± 13.2 mm/s	G*T: *p* = 0.636 G*: p* = 0.362 T*: p* = 0.248
	(6–156)	(6–46)	(7–56)	
NS sway velocity post	21.6 ± 17.4 mm/s	19.4 ± 7.4 mm/s	18.4 ± 14.5 mm/s	
	(7–58)	(11–35)	(7–59)	
FTS sway area pre^b^	3862 ± 3222 mm^2^	2583 ± 1427 mm^2^	2328 ± 1765 mm^2^	G*T: *p* = 0.694 G*: p* = 0.228 T*: p* = 0.578
	(603–9871)	(668–5015)	(608–6113)	
FTS sway area post^b^	2940 ± 2072 mm^2^	2556 ± 1585 mm^2^	2386 ± 1572 mm^2^	
	(796–7201)	(1174–6812)	(865–4792)	
FTS sway velocity pre^b^	84.1 ± 121.9 mm/s	65.9 ± 50.6 mm/s	57.0 ± 48.8 mm/s	G*T: *p* = 0.907 G*: p* = 0.552 T*: p* = 0.635
	(18–413)	(24–189)	(15–172)	
FTS sway velocity post^b^	72.5 ± 73.2 mm/s	50.3 ± 42.1 mm/s	59.2 ± 42.5 mm/s	
	(20–249)	(19–171)	(19–143)	
FTSec sway area pre^c^	3138 ± 3211 mm^2^	3797 ± 2479 mm^2^	3294 ± 2187 mm^2^	G*T: *p* = 0.567 G*: p* = 0.740 T*: p* = 0.850
	(718–10,114)	(1359–8491)	(1594–8413)	
FTSec sway area post^c^	3262 ± 2584 mm^2^	2844 ± 1872 mm^2^	4625 ± 4925 mm^2^	
	(1047–8714)	(816–6122)	(1267–17,036)	
FTSec sway velocity pre^c^	59.3 ± 40.4 mm/s	112.9 ± 77.5 mm/s	92.6 ± 67.9 mm/s	G*T: *p* = 0.686 G*: p* = 0.170 T*: p* = 0.682
	(22–138)	(29–256)	(37–211)	
FTSec sway velocity post^c^	61.4 ± 40.9 mm/s	84.8 ± 46.6 mm/s	98.3 ± 73.2 mm/s	
	(24–137)	(28–175)	(28–254)	

*Note*: *p*‐values highlighted in bold with asterisk represent significant two‐way ANOVA values with * uncorrected *p <* 0.05, ** Bonferroni‐corrected *p <* 0.05/n. Values highlighted in bold without asterisk represent significant changes between pre‐ and post‐training within one group (according to dependent samples *t*‐tests, *p*‐values reported in the Results section). G*T = interaction effect between group and time; G = main effect of group; T = main effect of time. TUG = Timed Up and Go test; ADL = Activities of Daily Living; SARA = Scale for the Assessment and Rating of Ataxia; ABC‐D = fall‐related self‐efficacy; FAHW = general habitual well‐being; NG = Normal Gait; BG = Backward Gait; NS = Natural Stance; FTS = Feet‐together Stance; FTSec = Feet‐together Stance with Eyes Closed.

^a^ *n* = 10 in group Train20 (one missing value).

^b^ *n* = 10 in group Train40 pre‐training.

^c^ *n* = 7 in control group pre‐ and post‐training, *n* = 9/*n* = 8 in group Train20 pre‐ and post‐training and *n* = 8/*n* = 9 in group Train40 pre‐ and post‐training.

Comparing Train20 + C and Train40 + C, no significant difference was found across measurement times for any of the dependent variables (all *p* ≥ 0.338, see Table S5), as well as no main effects of time (all *p* ≥ 0.434). Similar to the smaller groups, a generally lower balance confidence was found in group Train20 + C (*p*‐corr = 0.004).

In an exploratory approach, pre‐ and post‐training values were compared within each group. While no significant changes occurred in Train20, Train40 showed improvements in the primary outcome NG velocity (*p*‐unc = 0.030), in stride time (*p*‐unc = 0.050), double support (*p*‐unc = 0.033), and in BG step width (*p*‐unc = 0.011). The control group showed an improvement in NG step width (*p*‐unc = 0.045, see bold values in Table 2). Within the two extended training groups, only Train40 + C showed improvements in NG velocity (*p*‐unc = 0.025) and stride time (*p*‐unc = 0.027), and in the SARA‐score (1.5‐point reduction, *p*‐unc = 0.020, see Table S5).

**Figure 2:**
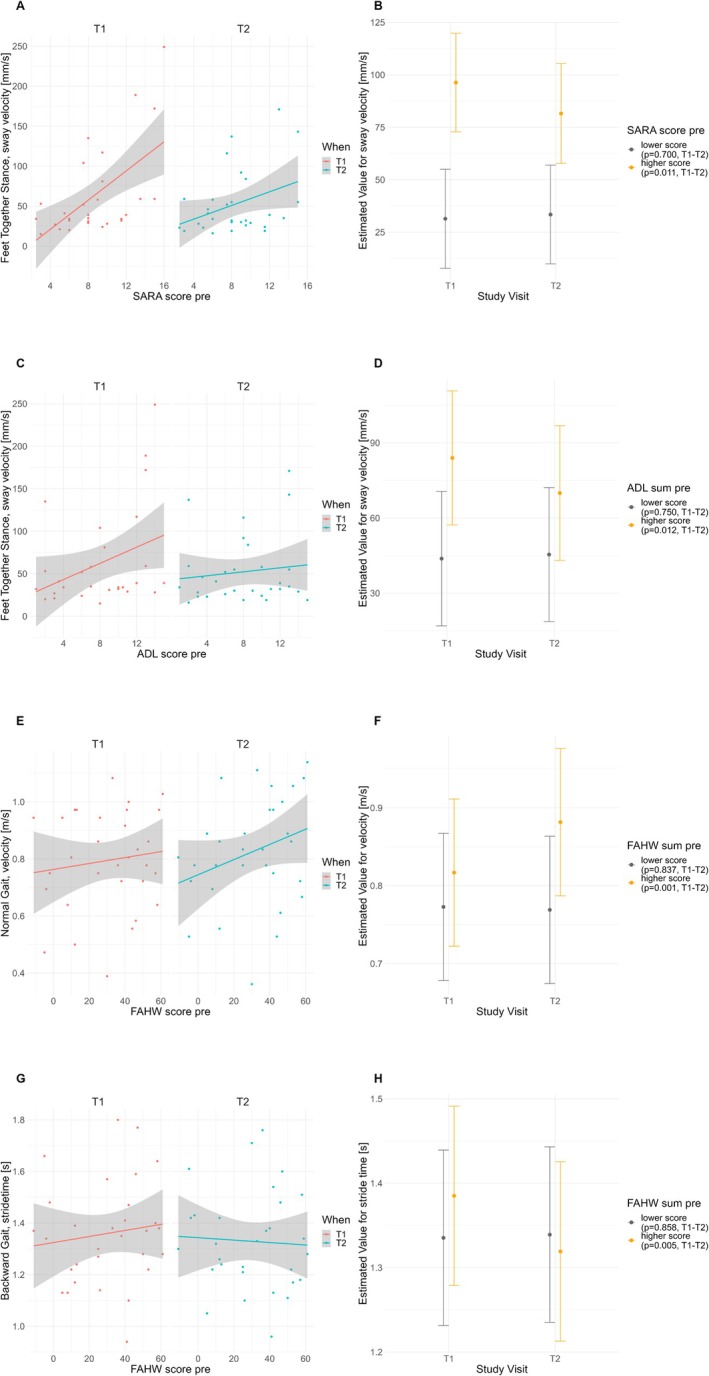
Effect of baseline clinical scores and study visit on gait and balance PerfO. Left panel (A, C, E, G): Linear relationship between baseline ClinRO/PRO (*x*‐axis) and PerfO scores pre‐ and post‐training (*y*‐axis) for variables with interactions. Right panel (B, D, F, H): Estimated marginal means with 95% confidence intervals for PerfO values, categorized by high and low ClinRO/PRO groups (orange and gray), pre‐ and post‐training.

*Interaction of ClinRO/PRO and PerfO*. To assess the impact of baseline ClinRO/PRO scores on changes in PerfO (T1 to T2) among all participants who underwent training, a linear mixed model was used. Significant “ClinRO/PRO × time” interactions were further analyzed, as this interaction suggests that initial clinical assessments may influence performance changes, aiding in identifying subgroups that respond differently to interventions. Significant interactions (see Fig. 2) were found between:

- SARA‐score and time, for sway velocity of FTS (*p*‐unc = 0.037*),
- ADL‐score and time, for sway velocity of FTS (*p*‐unc = 0.040*),
- FAHW‐score and time, for velocity of NG (*p*‐unc = 0.013*),
- FAHW‐score and time, for stride time of BG (*p*‐unc = 0.028*).

All other interactions did not reach statistical significance (all *p* ≥ 0.079). Several main effects for time and for ClinROs/PROs were found (Table S6).

Estimated marginal means for PerfO at T1 and T2 illustrated how ClinRO/PRO score levels influenced gait or balance at both time points (see Fig. 2, right panel). Pairwise contrasts revealed significant reductions in FTS sway velocity between T1 and T2 for those participants with higher SARA‐scores (mean score = 12.14, 14.8 mm/s reduction, *p*‐corr = 0.011) compared to those with lower scores (5.02, 3.0 mm/s increase, *p* = 0.700); for participants with higher ADL‐scores (mean score = 12.38, 14.1 mm/s reduction, *p*‐corr = 0.012) versus lower scores (3.97, 1.6 mm/s increase, *p* = 0.750); and significant increase in the primary outcome NG velocity and decrease in BG stride time between T1 and T2 for participants with higher FAHW‐scores (mean score = 51.60, 0.07 m/s increase and 0.07 s decrease, *p‐*corr = 0.001/0.005) versus lower scores (9.24, no change, *p* = 0.837/0.858).

## Discussion

The present study aimed to evaluate the effectiveness of video‐based training at home in improving gait and balance in people with hereditary cerebellar ataxia. In addition, it explored the influence of training session distribution, and the role of baseline clinical characteristics in determining training success. Our findings partially support our hypotheses, and highlight considerations for optimizing rehabilitation strategies, while aligning with key AGI criteria for gait and balance analyses.^22^ Furthermore, by demonstrating good to excellent reliability of all analyzed gait and balance variables and providing values for minimal detectable change, this study contributes to the ongoing effort of standardizing study protocols in clinical ataxia research.

Previous training studies, ranging from 4 to 30 weeks, with sessions 3–4 times per week, lasting 20–120 min, reported improvements in clinical scales as well as gait and balance outcomes following coordinative or balance training interventions,^8^, ^9^, ^11^, ^35^ while other studies presented mixed results.^12^, ^13^, ^14^, ^36^ These discrepancies highlight the need to determine the minimum effective requirements for training duration, intensity, and specific exercises, particularly for exclusive home‐based training programs. Contrary to our expectations and independent of the distribution of training sessions, our primary analysis revealed no significant effects of the three‐week video‐based training at home on gait and balance in people with mild to moderate cerebellar ataxia (mean SARA‐score of 8.4 ± 3.5). Nevertheless, including ClinRO as continuous predictors revealed that participants with varying levels of impairment obtained different outcomes (uncorrected level of significance). Participants with more severe impairment (as reflected in higher SARA and ADL scores) showed a significant improvement in balance (reduced sway velocity in feet‐together stance) after training. For these participants, the execution of this task already posed a greater challenge, but on the other hand also yielded more room for training‐related improvement. However, as this analysis assessed all participants together without control group comparison, it remains unclear whether the observed improvement can be attributed solely to the training effect. Notably, the more challenging tandem and single‐leg stance were excluded from analysis due to insufficient completion rates. Training effects in these tasks could have been of particular interest, even though rarely included in studies on cerebellar ataxia.^22^

As one of the few RCTs in the field of home‐based training in hereditary cerebellar ataxia,^11^, ^14^ our data indicate that the three‐week training may not have offered a sufficient volume (240 minutes) to achieve comparable outcomes, particularly for participants with mild/moderate ataxia. Discrepancies to studies reporting improvements after comparable training content^8^, ^9^, ^11^, ^35^ could be attributable to several factors: First, the lack of an overall training effect may be related to training intensity. While comparability to on‐site studies is limited, some studies employed similar training intensity and reported a significant training effect. However, these were not RCTs, and had smaller participant samples.^12^, ^35^ Second, due to the lack of standardization in most interventions, it remains unclear whether the observed effects in studies with supervised training—where the therapists often adapt exercises individually within a general framework—are attributable to the training program itself or to the influence of such individualized input. Future research should focus on developing standardized, clearly defined training protocols for people with hereditary cerebellar ataxia (or certain subgroups), allowing for better reproducibility and comparability, and enabling conclusions about which specific training parameters—such as a certain volume or frequency—are most effective. Third, while other interventions incorporated tasks in their training that resemble those assessed in the ClinRO and PerfO,^8^, ^35^ we used general coordination, mobility, and strength exercises without including any of the specific tasks assessed on‐site, to avoid any bias by a sole familiarization (practice effect). Fourth, the mean perceived difficulty of exercises in this study was rated 2.76 ± 1.22 out of 10, possibly indicating that the exercises were not challenging enough^37^ to provoke substantial improvements. However, when conducting home‐based training without supervision, it is crucial to select exercises that do not increase the risk of falling for people with ataxia, leading to a more conservative exercise selection. To address this limitation in future interventions, potential solutions would be to exclude exercises perceived as least challenging, to tailor the level of difficulty more specifically to patient subgroups (eg, regarding disease severity, age, use of assistant device) and/or to increase task complexity by introducing additional elements that enhance cognitive load, without necessarily increasing the physical difficulty of the task itself. This could involve adding a cognitive task (eg, simple counting tasks) or a parallel low‐effort motor task (eg, balancing a book with one hand while performing foot tapping).

We also explored training effects per group, although they are lacking direct comparison with a control group. Improvements (uncorrected level of significance) in NG variables, also including gait velocity as primary outcome of our RCT, were found in Train40 only, supported by similar effects in Train40 + C. Of note, in Train40 + C a significant improvement was found also for the SARA‐score (1.5‐point reduction, vs. 1‐point reduction in Train40). This is in line with previous studies, where reductions of 1.1–5.2 points were found after intensive, on‐site training programs^8^, ^11^, ^20^ and reductions of 0.6–1.9 points in home‐based programs.^12^, ^14^, ^36^ Mean progression rates in SCAs are 0.8–2.1 points/year,^38^ and a change of 1 SARA‐point can already be considered as meaningful,^39^ although it has been questioned whether the delta SARA‐score is an appropriate outcome from the perspective of those affected.^40^ Being the first study to directly compare two different training frequencies in people with mild/moderate degenerative hereditary cerebellar ataxia, our data suggest that longer, less frequent training sessions (eg, 2 × 40 minutes) are more effective. However, these preliminary findings should be further investigated in future studies, as it remains unclear whether session length, frequency, or the interaction between the two accounts for these effects.

Remarkably, although nearly all participants reported no depressive symptoms above the clinical cut‐off (except one scoring 19), those with higher well‐being scores (FAHW) at baseline demonstrated significant improvements (uncorrected significance) in gait velocity and stride time during normal and backward gait after training. Mental well‐being apparently not only enhances quality of life but may also be a relevant factor for optimizing the effectiveness of interventions to improve motor function. The results are consistent with a current study on people with Parkinson's Disease, which classified high responders and non‐responders of training and reported that high responders had the worst balance, slowest gait velocity, and lowest self‐perceived walking ability but also the highest balance confidence.^41^

### Limitations

The sample size has limited the statistical power of the study, potentially masking smaller but relevant effects, even though it was relatively high in comparison to similar studies.^12^, ^13^, ^14^ The large number of statistical tests and comparisons, which were not entirely independent, increased the likelihood of Type I errors. Additionally, we only measured gait at preferred speed. Current research recommends incorporating different gait speeds to provide greater challenge to cerebellar gait control.^22^, ^42^ Furthermore, participants adhered to their standard medical care routines, including physiotherapy, which may have introduced variability in treatment consistency. Last, we were unable to check how often or with what quality the training was performed, as this information was based solely on self‐reporting by the participants.

### Conclusions

This pilot RCT offers important insights into the effects of a 3‐week video‐based gait and balance training program at home. Although the training did not lead to general improvements in people with mild to moderate hereditary cerebellar ataxias, those with higher impairment and better mental well‐being experienced significant benefits, suggesting that these factors may influence the effectiveness of interventions, practice or adaptation processes. Furthermore, the data hint that it may be worth exploring the benefits of longer, less frequent training sessions. Our study provides valuable insights with regard to the overarching goal of defining effective, yet feasible and easily accessible training protocols.

## Author Roles

1. Research project: A. Conception, B. Organization, C. Execution, D. Resources;

2. Statistical Analysis: A. Design, B. Execution, C. Review and Critique;

3. Manuscript Preparation: A. Writing of the first draft, B. Review and Critique;

C.R.: 1A, 1B, 1C, 2A, 2B, 3A;

A.R.: 1C, 2C, 3B; NJ: 1C, 2C, 3B;

V.V.: 2C, 3B; JP: 1D, 2C, 3B;

H.J.: 1D, 2C, 3B;

D.T.: 1D, 2C, 3B;

A.T.: 1D, 2C, 3B;

D.B.: 1D, 3B;

C.B.: 1D, 2A, 2C, 3B;

A.S.: 1D, 2C, 3B;

K.A.: 1D, 2C, 3B;

M.M.: 1A, 1B, 1C, 1D, 2A, 2C, 3B.

## Disclosures

**Ethical Compliance Statement:** Written informed consent was obtained in agreement with the Declaration of Helsinki, and the study protocol was approved by the ethics committee of the Medical Faculty of the Heinrich Heine University Düsseldorf, Germany (2022–1994). The study was preregistered at ClinicalTrials.gov (NCT06617884). We confirm that we have read the Journal's position on issues involved in ethical publication and affirm that this work is consistent with those guidelines.

**Funding Sources and Conflict of Interest:** This study was supported by the Deutsche Heredo‐Ataxie‐Gesellschaft e. V. (DHAG), which provided the funding for patient compensation. The funding organization had no role in the design of the study, data collection, analysis, interpretation, or writing of the manuscript. This project has received funding from the European Union's Horizon Europe Programme under the Specific Grant Agreement No. 101147319 (EBRAINS 2.0 Project), the Helmholtz Association's Initiative and Networking Fund through the Helmholtz International BigBrain Analytics and Learning Laboratory (HIBALL) under the Helmholtz International Lab grant agreement InterLabs‐0015, and the Helmholtz Association joint lab “Supercomputing and Modeling for the Human Brain (SMHB).” The authors declare that there are no conflicts of interest regarding the publication of this paper and that they have no relevant financial or non‐financial interests to disclose.

**Financial Disclosures for the previous 12 months:** A.R. received funding from the Friedrich Ebert Foundation to support their doctoral research. A.T. held a position as a clinician scientist that was funded by the University Medicine Essen Clinician Scientist Academy (UMEA) and the German Research Foundation (Deutsche Forschungsgemeinschaft; DFG) at the time of the conduction of this study (grant no.: FU356/12‐2). A.S. received research support by the Deutsche Forschungsgemeinschaft and Brunhilde Moll Foundation. He received consulting fees and/or speaker honoraria from Abbott, Abbvie, Alexion, bsh medical communication, Novartis, GE Healthcare, and Zambon. M. M. was supported by the Deutsche Forschungsgemeinschaft (MI 709/2‐1) and received honoraria from Biogen, unrelated to this research.

## Supporting information


**TABLE S1.** Individual demographic and clinical characteristics of all included participants.
**TABLE S2.** Impaired vibration sense according to INAS classification in participants with pure and non‐pure cerebellar ataxia at baseline.
**TABLE S3.** Comparison of PerfO gait and balance variables between screening (T0) and baseline visit (T1) for the whole group.
**TABLE S4.** Comparison of demographic and clinical characteristics at baseline for the extended training groups.
**TABLE S5.** Comparison of longitudinal ClinRO, PRO and PerfO gait and balance variables between the extended training groups before and after training.
**TABLE S6.** Influence of ClinRO and time point on PerfO gait and balance variables.
**Supplementary Content**.
**Supplementary Methods:** Implementation details; Study design; Training program; Performance outcomes.
**Supplementary Results:** Time Interval Between T1 and T2; Baseline Standing and Gait Performance; Notable Patient Details; Stand Width Details; MDC, ICC, and Changes between T0 and T1 within the Whole Group; Baseline Comparison of the Two Extended Training Groups; Additional Analysis on Primary Outcome Gait Velocity; Analysis of the Training Effect within the Two Extended Training Groups; Interaction Details of ClinRO/PRO and PerfO; Impaired Proprioception/Vibration Sense According to INAS Classification.

## Data Availability

Anonymized data may be shared upon request to the corresponding or senior author from a qualified investigator for noncommercial use, subject to restrictions according to participant consent and data protection legislation. The data that support the findings of this study are available on request from the corresponding author. The data are not publicly available due to privacy or ethical restrictions.
