# Supplementary material for: Three‐Week Video‐ and Home‐Based Training Program for People with Ataxia: A Pilot Randomized Controlled Trial
Source: Mov Disord Clin Pract. 2025 Jul 14;12(12):2154–67. doi: 10.1002/mdc3.70225 (PMC12715367; doi:10.1002/mdc3.70225)
Supplement: Supplementary file 1 — TABLE S1. Individual demographic and clinical characteristics of all included participants. TABLE S2. Impaired vibration sense according to INAS classification in participants with pure and non‐pure cerebellar ataxia at baseline. TABLE S3. Comparison of PerfO gait and balance variables between screening (T0) and baseline visit (T1) for the whole group. TABLE S4. Comparison of demographic and clinical characteristics at baseline for the extended training groups. TABLE S5. Comparison of longitudinal ClinRO, PRO and PerfO gait and balance variables between the extended training groups before and after training. TABLE S6. Influence of ClinRO and time point on PerfO gait and balance variables. Supplementary Content. Supplementary Methods: Implementation details; Study design; Training program; Performance outcomes. Supplementary Results: Time Interval Between T1 and T2; Baseline Standing and Gait Performance; Notable Patient Details; Stand Width Details; MDC, ICC, and Changes between T0 and T1 within the Whole Group; Baseline Comparison of the Two Extended Training Groups; Additional Analysis on Primary Outcome Gait Velocity; Analysis of the Training Effect within the Two Extended Training Groups; Interaction Details of ClinRO/PRO and PerfO; Impaired Proprioception/Vibration Sense According to INAS Classification. [file MDC3-12-2154-s001.docx]

**Three-week Video- and Home-based Training Program for People with Ataxia: A Pilot Randomized Controlled Trial (Rentz et al., 2025)**

**Supplementary Methods:**

*Implementation Details.* Participants walked without shoes at a self-selected pace, while the examiner followed at a safe distance to mitigate any possible falls. During the natural stance, participants were asked to stand in a natural, stable position. In tandem stance, participants were allowed to decide which leg to put to the front, and in single-leg stance, they self-selected the foot to balance with.

*Study Design.* Following suggestions of recent studies^1,2^, we implemented a baseline measurement phase (baseline week) before the initial assessment to familiarize the participants with the assessment tasks and (technical) procedures.

*Training Program.* The training protocol was set up in close communication with an ataxia physiotherapy expert and based on sport-scientific knowledge of the author. All exercises were conducted as part of the additional exercise program, designed to complement, rather than replace, individual training or physiotherapy. The total training duration and session length were modeled after standard physiotherapy practices, which typically involve 6-12 sessions of approximately 20 minutes each, and based on literature, which suggests a drop in study compliance after three weeks^3^. The exercises focused on three key aspects of gait and balance improvement: coordination, strength, and mobility. Each video/training session consisted of all three of these key aspects. Exercises were repeated throughout the whole training program, and the difficulty level increased. For instance, protective steps^4^ were introduced in forward and backward directions during session 1/12, revisited in session 3/12, and supplemented with sideward protective steps in the same session. Participants were encouraged to perform these exercises in a relaxed and focused state to maximize effectiveness. For the group assigned to 2 x 40-minute training sessions, two video segments were combined for each session (e.g., segments 1+2 and 3+4) to streamline the exercise program. Coordination exercises were based on the key motor control and coordination skills^5^: Differentiation, orientation, reaction, balance, and rhythm ability. They were carried out in different variations and included fine motor exercises like synchronous and asynchronous head and finger movement, toe and foot arch movements, and eye movement tasks, as well as gross motor exercises like foot tappings, throwing a ball, clapping and stamping at certain displayed numbers, crawling, and clapping tasks, or combined the two aspects (walking on the spot while performing head/eye movements). Estimating times spans was used to train orientation ability, relevant for timing capabilities for motor and non-motor functions in which the cerebellum is involved^6^. Mobility exercises were included for the following reasons: With sufficient mobility, people can perform movements faster and more economically, which might also help to prevent falls. On the other hand, if mobility is restricted, this can lead to poor posture, increased risk of injury and limitations in everyday motor skills^7^. Exercises included stretching muscles and moving all joints through their full range of motion. Regarding strength training, the exercises were specifically chosen to target the muscles most involved in gait and balance, like gluteus maximus, gluteus medius, vasti, soleus, and gastrocnemius^8^. Some of the strength exercises were purely strength exercises, while others included coordination aspects: Stepping on a stack of books (while sitting), calf lifts and toe lifts, squats in front of a bed/table, weight shifting (while sitting), kneeling to lunge exercises, protective steps forward, backward, and sideward knee to elbow and leg raises (while sitting), deep sideward walks, and wall sits. More challenging coordination tasks were conducted sitting or lying on a mat, in order to minimize fall risk (e.g., weight shifting). Exercises across all three categories were progressively advanced from lying to sitting and then to standing positions during each session, whenever applicable.

In our study, we were unable to verify whether the at-home tasks and training sessions were performed correctly or consistently, which is a common limitation among home-based study interventions^9,10^. We designed a straightforward setup with minimal technical equipment and clear instructions, aiming to enhance participant adherence and minimize dropouts. This approach limited our ability to monitor the accuracy of the at-home activities. However, all participants confirmed completing the training and assessments.

*Longitudinal ClinRO and PRO (T0-T3):* INAS scores range from 0 (no non-ataxia symptoms) to 16 (maximum non-ataxia symptom load). The SARA yields a total score ranging from 0 (no signs of ataxia) to 40 (most severe ataxia), with higher scores indicating greater functional impairment. The TUG times the period required for the participant to stand up from a seated position, walk three meters, turn, return, and sit down. The FARS-ADL captures the degree of difficulty patients experience in daily tasks, with scores between 0 (no impairment in activities of daily living) and 36 (severe impairment). Lower FAHW‑scores indicate less well-being, and higher scores indicate greater well-being. ABC‑D scores range from 0% (no confidence) to 100% (full confidence).

*Performance Outcomes.* Stride time [s] describes the time between two heel contacts on the same side of the body, double support proportion [%] is the proportion of a gait cycle spent with both feet on the ground, the average velocity [m/s] is the average stride length divided by the average stride time, foot rotation [°] is the external rotation of the feet, step width [cm] is the lateral distance between the center of the left and right heel, and step width SD [cm] its standard deviation. In the stance tasks, which were used to assess balance, sway area [mm^2^] is the 95% confidence ellipse area of sway in the x-y-plane, and sway velocity [mm/s] is the total sway path divided by the total analysis time.

The TUG is not a ClinRO in a strict sense, but could also be regarded as a conventional PerfO.

**Supplementary Results:**

*Time Interval Between T1 and T2.* On average, 25.6±13.8 days passed between T1 and T2.

*Baseline Standing and Gait Performance.* At T1 (baseline) 100% of the participants were able to stand for 30 seconds in NS, 94% in FTS, 31% in TS, 81% in FTSec, and 13% in SS. 50% of participants were not able to perform TG without intermittently holding onto the wall.

**Supplementary Table 1:** Individual demographic and clinical characteristics of all included participants.

| **patient** | **genetic diagnose** | **age [years]** | **sex** | **Years of Onset** | **INAS [/16]** | **SARA [/40]** |
| --- | --- | --- | --- | --- | --- | --- |
| 1 | SCA6 | 66 | f | 13 | 1 | 13.5 |
| 2 | ADCAIII | 58 | f | 9 | 3 | 15 |
| 3 | ADCAIII | 55 | f | 5 | 2 | 10 |
| 4 | ATXN2/SCA2 | 40 | f | 3 | 0 | 7 |
| 5 | SCA1 | 65 | f | 19 | 4 | 8 |
| 6 | SCA3 | 60 | m | 4 | 2 | 8 |
| 7 | SCA6 | 64 | m | 12 | 3 | 8 |
| 8 | SCA6 | 66 | m | 1 | 3 | 12 |
| 9 | SCAR10 | 32 | f | 14 | 3 | 5.5 |
| 10 | SCAR10 | 34 | f | 8 | 3 | 9.5 |
| 11 | ATP6 mutation | 57 | m | 4 | 2 | 6 |
| 12 | CACNA1A | 58 | f | 36 | 2 | 7.5 |
| 13 | SCA6 | 70 | m | 10 | 4 | 16 |
| 14 | SCA6 | 64 | m | 4 | 1 | 9 |
| 15 | EA2 | 27 | m | 15 | 1 | 5 |
| 16 | CACNA1A | 55 | m | 23 | 2 | 8 |
| 17 | SCA27B | 40 | m | 6 | 2 | 4.5 |
| 18 | SCA6 | 55 | m | 3 | 2 | 15 |
| 19 | ADCAIII | 64 | f | 1 | 1 | 6 |
| 20 | SCA6 | 73 | m | 5 | 1 | 11.5 |
| 21 | SCA6 | 65 | f | 1 | 1 | 3 |
| 22 | SCA1 | 56 | f | 0 | 1 | 3 |
| 23 | SCA2 | 48 | m | 5 | 3 | 9.5 |
| 24 | SCA14 | 66 | m | 25 | 3 | 13 |
| 25 | SCA3 | 41 | f | 5 | 2 | 5.5 |
| 26 | SCA3 | 55 | f | 2 | 6 | 8 |
| 27 | SCA14 | 59 | f | 13 | 2 | 8 |
| 28 | SCA1 | 67 | f | 6 | 3 | 9.5 |
| 29 | SCA3 | 46 | m | 11 | 1 | 7.5 |
| 30 | SCA2 | 59 | m | 21 | 1 | 9 |
| 31 | SCA3 | 49 | m | 2 | 1 | 2.5 |
| 32 | SCA6 | 59 | f | 2 | 2 | 5.5 |
| 33 | SCA6 | 51 | m | 7 | 3 | 6 |
| 34 | SCA14 | 40 | m | 18 | 2 | 11.5 |

INAS = Inventory of Non-Ataxia Signs; SARA = Scale for the Assessment and Rating of Ataxia; ADCAIII = Autosomal Dominant Cerebellar Ataxia type III

*Notable Patient Details.* Patient 31 had an initial clinically assessed disease severity score of 3.5 (SARA) at screening for the study, but this dropped to a SARA score of 2.5 by the first study visit (classified as pre-ataxic according to the SARA scale authors). This reduction in score likely resulted from the weekly physiotherapy sessions he started in the interim. The Whole Group and group Train20+C included one subject with a moderately severe depression according to the PHQ-9 score^11^ (score of 19), and the same subject also indicated poor commitment to the training. Re-analyses after excluding this subject did not change the results substantially. One participant from the Whole Group scored very low on the MoCA test, with a score of 11 (most likely due to language barriers, participant was not a native German speaker), and was still included in the baseline measurements (this participant dropped out after T1). Another 15 of 34 participants only reached a MOCA score of 18 to 25 (normal score ≥ 26), corresponding to possible mild cognitive impairment^12^. The results may be biased, as tasks like drawing straight lines – which are typically impaired in ataxia – were required, although scores were relatively low in non-motor related areas as well.

*Stand Width Details.* The stand width (distance between the two heels) in the natural stance was 18.6 ± 6.0 cm, ranging from 8 cm to 29 cm.

*Impaired Proprioception/Vibration Sense According to INAS Classification.* In addition to cerebellar degeneration, impaired proprioception, which is frequently associated with impaired vibration sense, could contribute to gait and balance impairment and may interfere with a possible training effect. Impaired proprioception may occur more frequently in some types of hereditary ataxia (e.g., SCA3), often associated with polyneuropathy. We therefore compared the frequency of impaired vibration sense in participants with pure (hereditary ataxias commonly regarded as “pure” cerebellar ataxias (pureATX, e.g. SCA6, SCA14 etc.)) and non-pure hereditary ataxias. The distribution of severity of impaired vibration sense did not differ between the pure cerebellar and the non-pure cerebellar group (*p=*0.581 according to chi squared test).

**Supplementary Table 2:** Impaired vibration sense according to INAS classification in participants with pure and non-pure cerebellar ataxia at baseline.

| **INAS Vibration sense** | **all** | | **pure** | | **non-pure** | |
| --- | --- | --- | --- | --- | --- | --- |
|  | incidence | percent | incidence | percent | incidence | percent |
| not impaired (8/8) | 8 | 23.5 | 4 | 19.0 | 4 | 30.8 |
| not to mildly impaired | 3 | 8.8 | 3 | 14.3 | 0 | 0 |
| mildly impaired (>5/8) | 5 | 14.7 | 4 | 19.0 | 1 | 7.7 |
| mildly to moderately impaired | 6 | 17.6 | 3 | 14.3 | 3 | 23.1 |
| moderately impaired (2-5/8) | 4 | 11.8 | 3 | 14.3 | 1 | 7.7 |
| moderately to severely impaired | 3 | 8.8 | 1 | 4.8 | 2 | 15.4 |
| severely impaired (<2/8) | 5 | 14.7 | 3 | 14.3 | 2 | 15.4 |
| total | 34 | 100.0 | 21 | 100.0 | 13 | 100.0 |

INAS = Inventory of Non-Ataxia Signs

*MDC, ICC, and Changes between T0 and T1 within the Whole Group.* To confirm the stability and reliability of the gait and balance measures between screening and the baseline study visit (T0 and T1), we calculated the Minimal Detectable Change (MDC) and Intraclass Correlation Coefficient (ICC) values for each variable. Changes within the whole group in PerfO between T0 and T1 were identified using dependent samples t-tests.

**Supplementary Table 3:** Comparison of PerfO gait and balance variables between screening (T0) and baseline visit (T1) for the whole group.

| **Variable** | **Mean ± SD (Min.–Max.)** | | **n /**  **Sign.** | **MDC95 /**  **ICC** |
| --- | --- | --- | --- | --- |
|  | **T0** | **T1** |  |  |
| NG stride time | 1.19 ± 0.11 s | 1.19 ± 0.12 s | *n* = 33 | 0.10 |
|  | (0.95–1.42) | (0.95–1.51) | *p* = 0.974 | 0.89 |
| NG double supp. | 34.28 ± 6.48 % | 34.33 ± 5.20 % | *n* = 33 | 4.93 |
|  | (27.3–59.8) | (26.8–49.1) | *p* = 0.908 | 0.91 |
| NG velocity | 0.79 ± 0.16 m/s | 0.79 ± 0.16 m/s | *n* = 33 | 0.11 |
|  | (0.31–1.03) | (0.39–1.08) | *p* = 0.934 | 0.94 |
| NG foot rot. R | 13.78 ± 6.94 ° | 14.05 ± 6.98 ° | *n* = 33 | 3.32 |
|  | (-0.6–29.4) | (-0.1–29.5) | *p* = 0.376 | 0.97 |
| NG foot rot. L | 11.49 ± 6.93 ° | 11.30 ± 6.45 ° | *n* = 33 | 3.54 |
|  | (-0.1–31.5) | (-1.7–26.1) | *p* = 0.545 | 0.96 |
| NG step width | 16.64 ± 3.64 cm | 16.79 ± 3.91 cm | *n* = 33 | 2.67 |
|  | (10–25) | (9–26) | *p* = 0.530 | 0.93 |
| NG step width Var | 3.85 ± 1.12 cm | 3.79 ± 1.22 cm | *n* = 33 | 1.45 |
|  | (2–7) | (2–7) | *p* = 0.645 | 0.80 |
| BG stride time | 1.43 ± 0.29 s | 1.39 ± 0.24 s | *n* = 32 | 0.32 |
|  | (1.03–2.25) | (0.94–2.39) | *p* = 0.108 | 0.81 |
| BG double supp. | 49.89 ± 10.04 % | 48.20 ± 10.37 % | *n* = 32 | 9.15 |
|  | (33.2–76.8) | (30.3–74.8) | *p* = 0.051 | 0.89 |
| BG velocity | 0.35 ± 0.13 m/s | 0.37 ± 0.13 m/s | *n* = 32 | 0.13 |
|  | (0.08–0.61) | (0.11–0.64) | *p* = 0.067 | 0.87 |
| BG foot rot. R | 6.24 ± 6.03 ° | 6.53 ± 5.81 ° | *n* = 32 | 4.91 |
|  | (-9.6–24.2) | (-9.1–19.9) | *p* = 0.352 | 0.90 |
| BG foot rot. L | 3.26 ± 5.33 ° | 3.68 ± 6.01 ° | *n* = 32 | 3.89 |
|  | (-10.9–13.1) | (-13.6–14.8) | *p* = 0.422 | 0.94 |
| BG step width | 24.90 ± 4.15 cm | 25.16 ± 4.41 cm | *n* = 32 | 3.24 |
|  | (16–34) | (15–35) | *p* = 0.402 | 0.92 |
| BG step width Var | 2.88 ± 1.31 cm | 3.00 ± 1.30 cm | *n* = 32 | 1.77 |
|  | (2–8) | (1–7) | *p* = 0.442 | 0.76 |
| NS sway area | 929 ± 810 mm^2^ | 942 ± 1001 mm^2^ | *n* = 32/33 | 1400 |
|  | (100–3272) | (77–4045) | *p* = 0.550 | 0.69 |
| NS sway velocity | 27.0 ± 26.8 mm/s | 26.1 ± 27.0 mm/s | *n* = 32/33 | 21.4 |
|  | (5–137) | (6–156) | *p* = 0.948 | 0.92 |
| FTS sway area | 3053 ± 2152 mm^2^ | 3109 ± 2483 mm^2^ | *n* = 33/31 | 3031 |
|  | (117–8580) | (603–9871) | *p* = 0.695 | 0.77 |
| FTS sway velocity | 59.5 ± 51.1 mm/s | 68.7 ± 77.9 mm/s | *n* = 33/31 | 64.1 |
|  | (15–272) | (15–413) | *p* = 0.227 | 0.87 |
| FTSec sway area | 3628 ± 2403 mm^2^ | 3605 ± 2609 mm^2^ | *n* = 20/25 | 3605 |
|  | (822–9003) | (718–10114) | *p* = 0.204 | 0.73 |
| FTSec sway velocity | 75.2 ± 45.0 mm/s | 90.5 ± 65.0 mm/s | *n* = 20/25 | 55.2 |
|  | (22–213) | (22–256) | *p* = 0.794 | 0.88 |

NG = Normal Gait; BG = Backward Gait; NS = Natural Stance; FTS = Feet Together Stance; FTSec = Feet Together Stance with Eyes Closed; Sign. = *p*-values according to dependent samples t-tests within the whole group between T0 and T1; MDC95 = Minimal Detectable Change at a 95 % confidence level; ICC = Intraclass Correlation Coefficient (test-retest reliability)

*Baseline Comparison of the Two Extended Training Groups.* There was no difference between Train20+C (n=15, 8 female, 0 left-handed, disease duration of 10.4±10.4 years, SARA 8.9±3.3, 10 pureATX) and Train40+C (n=16, 6 female, 1 left-handed, disease duration of 7.9±5.6 years, SARA 8.3±3.9, 10 pureATX) in the distribution of sex, handedness, disease duration, SARA score, and number of pure cerebellar ataxias (p≥0.361). The two training groups did not differ significantly in age, height or weight (*p*≥0.622), but participants of Train40+C had a higher number of years of education (*p*‑unc*=*0.004, see Supplementary Table 4). The two training groups did not differ significantly in the amount of physical/sport activity per week and per day, the amount of physiotherapy, occupational and speech therapy per week, the estimated number of falls within the past six month and past week, the depression score or the cognitive assessment (*p*≥0.232). The groups also showed no differences in the PGIC questionnaire (*p*≥0.061) and no different initial ClinRO and PRO scores, except for the ABC-D score, where balance confidence was higher in Train40+C (67.44±17.54) compared to Train20+C (55.50±14.64, *p*‑unc=0.050).

**Supplementary Table 4:** Comparison of demographic and clinical characteristics at baseline for the extended training groups.

|  | **Whole Group** | **Train20+C** | **Train40+C** | **Group comparison** |
| --- | --- | --- | --- | --- |
| **Age [years]** | 54.8 ± 11.5  (27 - 73)  n = 34 | 54.7 ± 11.3  (32 - 66)  n = 15 | 56.8 ± 12.0  (27 - 73)  n = 16 | *p* = 0.622 |
| **Height [cm]** | 173.1 ± 8.1  (157 – 190)  n = 34 | 173.0 ± 9.5  (157 – 190)  n = 15 | 173.1 ± 6.8  (163 – 187)  n = 16 | *p* = 0.983 |
| **Weight [kg]** | 73.1 ± 14.5  (50 – 108)  n = 29 | 73.0 ± 16.8  (50 – 97)  n = 14 | 74.4 ± 12.3  (58 – 108)  n = 14 | *p* = 0.809 |
| **Disease duration [years]** | 8.8 ± 8.3  (0 - 36)  n = 32 | 10.4 ± 10.4  (0 - 36)  n = 15 | 6.7 ± 4.5  (1 - 15)  n = 14 | *p* = 0.412 |
| **PHQ-9 score [/27]** | 6.8 ± 4.0  (1 - 19)  n = 34 | 7.2 ± 4.3  (2 - 19)  n = 15 | 6.1 ± 3.8  (1 - 12)  n = 16 | *p* = 0.440 |
| **MOCA score [/30]** | 25.0 ± 3.9  (11 – 30)  n = 34 | 24.9 ± 3.6  (18 - 30)  n = 15 | 26.3 ± 2.3  (21 - 29)  n = 16 | *p* = 0.210 |
| **Education  [years]** | 15.5 ± 2.9  (11 - 23)  n = 34 | 14.0 ± 2.1  (11 - 18)  n = 15 | 17.0 ± 3.2  (12 - 23)  n = 16 | ***p* = 0.004*** |
| **Sport per week [days]** | 4.2 ± 2.2  (0 - 7)  n = 34 | 4.3 ± 1.8  (1 - 7)  n = 15 | 4.3 ± 2.7  (0 - 7)  n = 16 | *p* = 0.988 |
| **Sport per day [hours]** | 1.6 ± 1.2  (0 - 6)  n = 33 | 2.0 ± 1.5  (0.5 - 6)  n = 14 | 1.2 ± 0.8  (0 – 2.5)  n = 16 | *p* = 0.112 |
| **Physiotherapy per week [amount]** | 1.4 ± 0.8  (0 - 3)  n = 34 | 1.6 ± 0.7  (0 - 3)  n = 15 | 1.2 ± 0.8  (0 - 2  n = 16 | *p* = 0.156 |
| **Occupational therapy per week [amount]** | 0.4 ± 0.6  (0 - 2)  n = 34 | 0.4 ± 0.6  (0 - 2)  n = 15 | 0.3 ± 0.6  (0 - 2)  n = 16 | *p* = 0.587 |
| **Speech therapy per week [amount]** | 0.4 ± 0.8  (0 - 3)  n = 34 | 0.4 ± 0.8  (0 - 3)  n = 15 | 0.2 ± 0.4  (0 - 1)  n = 16 | *p* = 0.311 |
| **Falls within 6 months [amount]** | 3.4 ± 7.8  (0 - 40)  n = 30 | 2.8 ±4.7  (0 - 18)  n = 14 | 1.5 ± 2.1  (0 - 6)  n = 13 | *p* = 0.392 |
| **Almost falls within 6 months [amount]** | 19.0 ± 66.1  (0 - 365)  n = 30 | 35.0 ± 95.7  (0 - 365)  n = 14 | 6.2 ± 8.2  (0 - 25)  n = 13 | *p* = 0.290 |
| **Falls within 1 week [amount]** | 0.5 ± 1.9  (0 - 10)  n = 31 | 0.3 ± 0.8  (0 - 3)  n = 15 | 0.1 ± 0.3  (0 - 1)  n = 13 | *p* = 0.423 |
| **Almost falls within 1 week [amount]** | 1.9 ± 5.0  (0 - 20)  n = 31 | 2.0 ± 4.9  (0 - 18)  n = 15 | 2.2 ± 5.8  (0 - 20)  n = 13 | *p* = 0.940 |

*p*-values highlighted in bold with asterisk represent significant values with * uncorrected *p<*0.05 (independent sample t-tests between the two extended training groups). Disease duration = Years since disease onset; Education = Educational level measured in years of Education; Sport per week/day = Amount of physical/sport activity perceived as demanding, per week or per day; PHQ-9 = depression module of the Patient Health Questionnaire; MOCA = cognitive impairment according to the Montreal Cognitive Assessment Test

*Additional Analysis on Primary Outcome Gait Velocity.* We conducted independent samples t-tests to compare the improvements in gait velocity (difference of T2 minus T1) between groups, calculating effect sizes (Cohen's *d*) for the primary outcome. Specifically, we analyzed the differences between the control group vs. Train20, control group vs. Train40, and Train20 vs. Train40. These effect sizes may serve as useful metrics for future study planning and sample size estimation in similar contexts.

Between-group differences in gait velocity improvements were assessed using independent samples t-tests and associated effect sizes (Cohen’s *d*) with 95% confidence intervals (CIs).

- Control group vs. Train20: The analysis revealed no significant difference in gait velocity improvements (*p*=0.687), with a small effect size (*d*=0.184; 95% CI: −0.702, 1.064).
- Control group vs. Train40: Similarly, no significant difference was observed (*p*=0.269), with a medium effect size favoring Train40 (*d*=−0.512; 95% CI: −1.402, 0.391).
- Train20 vs. Train40: While this comparison did not reach significance (*p*=0.133), a large effect size (*d*=−0.667; 95% CI: −1.520, 0.201) was observed, favoring Train40.

Effect sizes (Cohen’s *d*) were interpreted according to established guidelines: 0.1-0.2 as small, 0.3-0.4 as medium, 0.5-0.7 as large, and >0.7 as very large^13^.

*Analysis of the Training Effect within the Two Extended Training Groups.* The following table presents descriptive data for clinical variables, as well as gait and balance measures, specifically for the two extended training groups (Train20+C, Train40+C). Additionally, the table includes the results of the ANOVA interaction and main effects, highlighting any significant differences between the groups in these variables.

**Supplementary Table 5:** Comparison of longitudinal ClinRO, PRO and PerfO gait and balance variables between the extended training groups before and after training.

| **Variable** | **Mean ± SD  (Min.–Max.)** | | **Interaction Effect Main Effect Group Main Effect Time** |
| --- | --- | --- | --- |
|  | **Train20+C** | **Train40+C** |  |
| SARA score pre | 8.9 ± 3.3 | **8.3 ± 3.9** | *p* = 0.425  *p* = 0.158  *p* = 0.369 |
|  | (3–15) | **(2.5–16)** |  |
| SARA score post | 8.8 ± 2.2 | **6.8 ± 4.1** |  |
|  | (5.5–13.5) | **(0–16)** |  |
| ADL score pre | 7.8 ± 4.2 | 8.6 ± 4.4 | *p* = 0.579  *p* = 0.859  *p* = 0.766 |
|  | (1–13) | (2–15) |  |
| ADL score post | 8.1 ± 4.1 | 7.7 ± 4.3 |  |
|  | (1–14) | (0–15) |  |
| TUG time pre | 12.6 ± 5.4s | 11.0 ± 3.4s | *p* = 0.747  *p* = 0.089  *p* = 0.962 |
|  | (6.2–26.5) | (4.7–17.1) |  |
| TUG time post | 13.0 ± 5.1s | 10.7 ± 3.7s |  |
|  | (6.4–24.3) | (4.5–17.4) |  |
| ABCD score pre | 55.50 ± 14.64 | 67.44 ± 17.54 | *p* = 0.993  ***p* = 0.004****  *p* = 0.844 |
|  | (31.6-77.8) | (30.0-89.1) |  |
| ABCD score post | 56.32 ± 13.67 | 68.19 ± 15.43 |  |
|  | (34.0-81.0) | (36.0-94.0) |  |
| FAHW score pre | 30.20 ± 24.98 | 30.63 ± 18.15 | *p* = 0.580  *p* = 0.537  *p* = 0.912 |
|  | (-11-61) | (5-59) |  |
| FAHW score post | 27.43 ± 28.85 | 34.38 ± 18.65 |  |
|  | (-15-73) | (5-64) |  |
| NG stride time pre | 1.18 ± 0.15 s | **1.19 ± 0.11 s** | *p* = 0.601  *p* = 0.589  *p* = 0.402 |
|  | (0.95–1.51) | **(1.01–1.37)** |  |
| NG stride time post | 1.18 ± 0.14 s | **1.16 ± 0.10 s** |  |
|  | (0.92–1.46) | **(0.99–1.32)** |  |
| NG double supp. pre | 35.50 ± 7.48 % | 33.13 ± 2.97 % | *p* = 0.981  *p* = 0.113  *p* = 0.685 |
|  | (26.8–52.3) | (27.5–38.1) |  |
| NG double supp. post | 34.93 ± 8.16 % | 32.49 ± 3.26 % |  |
|  | (26.1–57.0) | (27.6–38.3) |  |
| NG velocity pre | 0.77 ± 0.21 m/s | **0.82 ± 0.14 m/s** | *p* = 0.689  *p* = 0.162  *p* = 0.516 |
|  | (0.39–1.08) | **(0.50–0.97)** |  |
| NG velocity post | 0.78 ± 0.23 m/s | **0.87 ± 0.15 m/s** |  |
|  | (0.36–1.14) | **(0.56–1.08)** |  |
| NG foot rot. pre | 12.3 ± 6.9 ° | 13.6 ± 5.1 ° | *p* = 0.989  *p* = 0.863  *p* = 0.402 |
|  | (3.2–24.0) | (4.0–24.0) |  |
| NG foot rot. post | 12.6 ± 7.1 ° | 13.8 ± 5.7 ° |  |
|  | (2.3–25.0) | (5.6–26.8) |  |
| NG step width pre | 17.82 ± 3.82 cm | 17.36 ± 4.39 cm | *p* = 0.676  *p* = 0.832  *p* = 0.402 |
|  | (11–23) | (12–26) |  |
| NG step width post | 18.55 ± 2.98 cm | 16.09 ± 3.51 cm |  |
|  | (13–23) | (12–25) |  |
| NG step width SD pre | 3.73 ± 1.39 cm | 3.88 ± 0.87 cm | *p* = 0.848  *p* = 0.524  *p* = 0.402 |
|  | (2–7) | (3–6) |  |
| NG step width SD post | 3.60 ± 1.24 cm | 3.63 ± 1.15 cm |  |
|  | (2–6) | (2–6) |  |
| BG stride time pre | 1.39 ± 0.18 s | 1.33 ± 0.22 s | *p* = 0.861  *p* = 0.558  *p* = 0.402 |
|  | (1.10–1.66) | (0.94–1.80) |  |
| BG stride time post | 1.35 ± 0.19 s | 1.31 ± 0.20 s |  |
|  | (1.10–1.71) | (0.96–1.76) |  |
| BG double supp. pre | 49.70 ± 11.32 % | 46.51 ± 8.89 % | *p* = 0.818  *p* = 0.298  *p* = 0.801 |
|  | (38.0–74.8) | (30.3–62.6) |  |
| BG double supp. post | 48.46 ± 10.67 % | 46.41 ± 7.58 % |  |
|  | (37.0–75.6) | (32.5–60.7) |  |
| BG velocity pre | 0.37 ± 0.16 m/s | 0.41 ± 0.14 m/s | *p* = 0.846  *p* = 0.686  *p* = 0.402 |
|  | (0.11–0.67) | (0.19–0.69) |  |
| BG velocity post | 0.39 ± 0.17 m/s | 0.42 ± 0.13 m/s |  |
|  | (0.11–0.72) | (0.22–0.75) |  |
| BG foot rot. pre | 4.3 ± 4.2 ° | 5.8 ± 5.3 ° | *p* = 0.811  *p* = 0.169  *p* = 0.809 |
|  | (-3.1–11.4) | (-5.5–14.8) |  |
| BG foot rot. post | 4.3 ± 4.4 ° | 6.4 ± 5.5 ° |  |
|  | (3.9–10.2) | (-4.1–16.6) |  |
| BG step width pre | 25.14 ± 4.00 cm | 25.06 ± 4.22 cm | *p* = 0.929  *p* = 0.987  *p* = 0.976 |
|  | (18–31) | (17–35) |  |
| BG step width post | 25.07 ± 4.10 cm | 25.19 ± 4.62 cm |  |
|  | (18–31) | (16–34) |  |
| BG step width SD pre | 2.86 ± 1.10 cm | 3.06 ± 1.44 cm | *p* = 0.518  *p* = 1.000  *p* = 0.833 |
|  | (2–6) | (1–6) |  |
| BG step width SD post | 3.14 ± 1.23 cm | 2.94 ± 1.06 cm |  |
|  | (2–6) | (1–5) |  |
| NS sway area pre | 925 ± 804 mm^2^ | 707 ± 797 mm^2^ | *p* = 0.390  *p* = 0.059  *p* = 0.855 |
|  | (208–2854) | (86–3036) |  |
| NS sway area post | 1073 ± 1125 mm^2^ | 495 ± 379 mm^2^ |  |
|  | (252–3700) | (81–1275) |  |
| NS sway velocity pre | 22.7 ± 12.8 mm/s | 20.1 ± 15.7 mm/s | *p* = 0.982  *p* = 0.506  *p* = 0.968 |
|  | (6–46) | (7–58) |  |
| NS sway velocity post | 22.6 ± 14.6 mm/s | 19.8 ± 19.3 mm/s |  |
|  | (11–70) | (4–75) |  |
| FTS sway area pre | 2848 ± 1775 mm^2^ | 2243 ± 1635 mm^2^ | *p* = 0.540  *p* = 0.069  *p* = 0.781 |
|  | (668–7201) | (608–6113) |  |
| FTS sway area post | 3299 ± 2558 mm^2^ | 2081 ± 1454 mm^2^ |  |
|  | (1086–10370) | (557–4792) |  |
| FTS sway velocity pre | 65.1 ± 48.2 mm/s | 62.1 ± 66.3 mm/s | *p* = 0.890  *p* = 0.719  *p* = 0.388 |
|  | (24–189) | (15–249) |  |
| FTS sway velocity post | 55.6 ± 42.4 mm/s | 48.9 ± 40.1 mm/s |  |
|  | (19–171) | (16–143) |  |
| FTSec sway area pre | 3656 ± 2250 mm^2^ | 2869 ± 1939 mm^2^ | *p* = 0.338  *p* = 0.962  *p* = 0.701 |
|  | (1359–8491) | (1047–8413) |  |
| FTSec sway area post | 3090 ± 1948 mm^2^ | 3943 ± 4241 mm^2^ |  |
|  | (816–6122) | (666–17036) |  |
| FTSec sway velocity pre | 104.8 ± 72.4 mm/s | 74.8 ± 61.1 mm/s | *p* = 0.511  *p* = 0.333  *p* = 0.668 |
|  | (29–256) | (24–211) |  |
| FTSec sway velocity post | 83.4 ± 42.5 mm/s | 78.0 ± 68.1 mm/s |  |
|  | (28–175) | (20–254) |  |

*p*-values highlighted in bold with asterisk represent significant two-way ANOVA values with ** Bonferroni-corrected *p<*0.05/n. Values highlighted in bold without asterisk represent significant changes between pre- and post-training within one group (according to dependent samples t-tests, p-values reported in the Results section). NG = Normal Gait; BG = Backward Gait; NS = Natural Stance; FTS = Feet Together Stance; FTSec = Feet Together Stance with Eyes Closed

*Interaction Details of ClinRO/PRO and PerfO.* The following table presents the details of Analysis 5, which assessed the influence of clinical scores on changes in gait and balance variables across the intervention phase. The model included fixed effects for clinical scores and time point (T1, T2), as well as their interaction. A random intercept for individual subjects was added to account for variability in baseline levels. The table highlights significant associations between clinical scores and gait or balance outcomes, focusing on the main effects and interactions.

**Supplementary Table 6:** Influence of ClinRO and time point on PerfO gait and balance variables.

| **Clinical variable** | **Dependent variable** | **Interaction Effect** | **Main Effect Clin. Var.** | **Main Effect Time** |
| --- | --- | --- | --- | --- |
| SARA | NG stride time | *p* = 0.320 | *p* = 0.975 | *p* = 0.787 |
|  | NG double supp. | *p* = 0.664 | ***p* = 0.049*** | *p* = 0.659 |
|  | NG velocity | *p* = 0.176 | ***p* = 0.012*** | *p* = 0.890 |
|  | NG foot rot. | *p* = 0.757 | *p* = 0.482 | *p* = 0.478 |
|  | NG step width | *p* = 0.383 | ***p* < 0.001**** | *p* = 0.545 |
|  | NG step width SD | *p* = 0.402 | *p* = 0.286 | *p* = 0.169 |
|  | BG stride time | *p* = 0.198 | *p* = 0.928 | *p* = 0.633 |
|  | BG double supp. | *p* = 0.284 | ***p* = 0.011*** | *p* = 0.606 |
|  | BG velocity | *p* = 0.524 | ***p* = 0.035*** | *p* = 0.953 |
|  | BG foot rot. | *p* = 0.683 | *p* = 0.734 | *p* = 0.450 |
|  | BG step width | *p* = 0.555 | ***p* = 0.022*** | *p* = 0.611 |
|  | BG step width SD | *p* = 0.991 | *p* = 0.915 | *p* = 0.866 |
|  | NS sway area | *p* = 0.392 | *p* = 0.826 | *p* = 0.498 |
|  | NS sway velocity | *p* = 0.503 | *p* = 0.171 | *p* = 0.512 |
|  | FTS sway area | *p* = 0.135 | ***p* = 0.040*** | *p* = 0.106 |
|  | FTS sway velocity | ***p* = 0.037*** | ***p* < 0.001**** | *p* = 0.166 |
|  | FTSec sway area | *p* = 0.228 | *p* = 0.624 | *p* = 0.389 |
|  | FTSec sway velocity | *p* = 0.481 | *p* = 0.391 | *p* = 0.864 |
| ADL | NG stride time | *p* = 0.212 | *p* = 0.457 | *p* = 0.736 |
|  | NG double supp. | *p* = 0.991 | ***p* = 0.006**** | *p* = 0.583 |
|  | NG velocity | *p* = 0.210 | ***p* = 0.005**** | *p* = 0.884 |
|  | NG foot rot. | *p* = 0.371 | *p* = 0.589 | *p* = 0.194 |
|  | NG step width | *p* = 0.079 | ***p* = 0.003**** | *p* = 0.182 |
|  | NG step width SD | *p* = 0.086 | *p* = 0.112 | ***p* = 0.025*** |
|  | BG stride time | *p* = 0.125 | *p* = 0.462 | *p* = 0.604 |
|  | BG double supp. | *p* = 0.193 | ***p* = 0.004*** | *p* = 0.552 |
|  | BG velocity | *p* = 0.164 | ***p* = 0.006*** | *p* = 0.544 |
|  | BG foot rot. | *p* = 0.663 | *p* = 0.690 | *p* = 0.403 |
|  | BG step width | *p* = 0.733 | ***p* = 0.020*** | *p* = 0.795 |
|  | BG step width SD | *p* = 0.434 | *p* = 0.241 | *p* = 0.611 |
|  | NS sway area | *p* = 0.578 | *p* = 0.874 | *p* = 0.719 |
|  | NS sway velocity | *p* = 0.978 | *p* = 0.757 | *p* = 0.945 |
|  | FTS sway area | *p* = 0.772 | *p* = 0.267 | *p* = 0.600 |
|  | FTS sway velocity | ***p* = 0.040*** | ***p* = 0.039*** | *p* = 0.260 |
|  | FTSec sway area | *p* = 0.486 | *p* = 0.427 | *p* = 0.785 |
|  | FTSec sway velocity | *p* = 0.175 | *p* = 0.210 | *p* = 0.935 |

| TUG | NG stride time | *p* = 0.885 | *p* = 0.653 | *p* = 0.647 |
| --- | --- | --- | --- | --- |
|  | NG double supp. | *p* = 0.456 | ***p* < 0.001**** | *p* = 0.267 |
|  | NG velocity | *p* = 0.906 | ***p* < 0.001**** | *p* = 0.531 |
|  | NG foot rot. | *p* = 0.108 | *p* = 0.229 | *p* = 0.059 |
|  | NG step width | *p* = 0.928 | *p* = 0.053 | *p* = 0.787 |
|  | NG step width SD | *p* = 0.359 | ***p* = 0.002**** | *p* = 0.158 |
|  | BG stride time | *p* = 0.626 | *p* = 0.056 | *p* = 0.855 |
|  | BG double supp. | *p* = 0.164 | ***p* < 0.001**** | *p* = 0.379 |
|  | BG velocity | *p* = 0.223 | ***p* < 0.001**** | *p* = 0.507 |
|  | BG foot rot. | *p* = 0.343 | *p* = 0.271 | *p* = 0.219 |
|  | BG step width | *p* = 0.447 | *p* = 0.074 | *p* = 0.496 |
|  | BG step width SD | *p* = 0.460 | *p* = 0.054 | *p* = 0.583 |
|  | NS sway area | *p* = 0.762 | *p* = 0.666 | *p* = 0.698 |
|  | NS sway velocity | *p* = 0.395 | *p* = 0.535 | *p* = 0.445 |
|  | FTS sway area | *p* = 0.352 | *p* = 0.332 | *p* = 0.502 |
|  | FTS sway velocity | *p* = 0.799 | *p* = 0.399 | *p* = 0.749 |
|  | FTSec sway area | *p* = 0.234 | *p* = 0.764 | *p* = 0.340 |
|  | FTSec sway velocity | *p* = 0.163 | *p* = 0.414 | *p* = 0.474 |
| FAHW | NG stride time | *p* = 0.187 | *p* = 0.417 | *p* = 0.913 |
|  | NG double supp. | *p* = 0.667 | *p* = 0.564 | *p* = 0.747 |
|  | NG velocity | ***p* = 0.013*** | *p* = 0.508 | *p* = 0.414 |
|  | NG foot rot. | *p* = 0.097 | *p* = 0.566 | ***p* = 0.046*** |
|  | NG step width | *p* = 0.944 | *p* = 0.519 | *p* = 0.806 |
|  | NG step width SD | *p* = 0.641 | *p* = 0.640 | *p* = 0.200 |
|  | BG stride time | ***p* = 0.028*** | *p* = 0.499 | *p* = 0.471 |
|  | BG double supp. | *p* = 0.974 | *p* = 0.500 | *p* = 0.497 |
|  | BG velocity | *p* = 0.231 | *p* = 0.693 | *p* = 0.857 |
|  | BG foot rot. | *p* = 0.301 | *p* = 0.621 | *p* = 0.159 |
|  | BG step width | *p* = 0.897 | *p* = 0.748 | *p* = 0.873 |
|  | BG step width SD | *p* = 0.671 | *p* = 0.340 | *p* = 0.560 |
|  | NS sway area | *p* = 0.148 | ***p* = 0.031*** | *p* = 0.304 |
|  | NS sway velocity | *p* = 0.911 | ***p* = 0.018*** | *p* = 0.883 |
|  | FTS sway area | *p* = 0.093 | *p* = 0.193 | *p* = 0.082 |
|  | FTS sway velocity | *p* = 0.275 | *p* = 0.236 | *p* = 0.086 |
|  | FTSec sway area | *p* = 0.302 | *p* = 0.141 | *p* = 0.682 |
|  | FTSec sway velocity | *p* = 0.759 | ***p* = 0.046*** | *p* = 0.293 |
| ABC-D | NG stride time | *p* = 0.835 | *p* = 0.718 | *p* = 0.808 |
|  | NG double supp. | *p* = 0.542 | ***p* = 0.008*** | *p* = 0.369 |
|  | NG velocity | *p* = 0.693 | ***p* = 0.002*** | *p* = 0.353 |
|  | NG foot rot. | *p* = 0.242 | *p* = 0.227 | *p* = 0.403 |
|  | NG step width | *p* = 0.760 | ***p* = 0.003*** | *p* = 0.664 |
|  | NG step width SD | *p* = 0.810 | ***p* = 0.013*** | *p* = 0.849 |
|  | BG stride time | *p* = 0.641 | *p* = 0.867 | *p* = 0.363 |
|  | BG double supp. | *p* = 0.487 | ***p* = 0.020*** | *p* = 0.327 |
|  | BG velocity | *p* = 0.382 | ***p* = 0.004*** | *p* = 0.235 |
|  | BG foot rot. | *p* = 0.900 | *p* = 0.783 | *p* = 0.711 |
|  | BG step width | *p* = 0.602 | ***p* = 0.015*** | *p* = 0.597 |
|  | BG step width SD | *p* = 0.593 | *p* = 0.123 | *p* = 0.535 |
|  | NS sway area | *p* = 0.864 | *p* = 0.954 | *p* = 0.808 |
|  | NS sway velocity | *p* = 0.577 | *p* = 0.286 | *p* = 0.573 |
|  | FTS sway area | *p* = 0.528 | *p* = 0.333 | *p* = 0.443 |
|  | FTS sway velocity | *p* = 0.446 | *p* = 0.064 | *p* = 0.253 |
|  | FTSec sway area | *p* = 0.249 | *p* = 0.746 | *p* = 0.199 |
|  | FTSec sway velocity | *p* = 0.556 | *p* = 0.422 | *p* = 0.276 |

Results of the linear mixed model including the fixed effects of clinical scores and time point (T1, T2), as well as their interaction (PerfO ~ ClinRO/PRO * time_point + (1 | ID)). *p*-values highlighted in bold represent significant values with * uncorrected *p<*0.05, ** Bonferroni-corrected *p<*0.05/18. TUG = Timed Up and Go test; SARA = Scale for the Assessment and Rating of Ataxia; ADL = Activities of Daily Living; FAHW = general habitual well-being; ABC‑D = balance confidence; NG = Normal Gait; BG = Backward Gait; NS = Natural Stance; FTS = Feet Together Stance; FTSec = Feet Together Stance with Eyes Closed

References

1. Meyer C, Killeen T, Lörincz L, et al. Repeated assessment of key clinical walking measures can induce confounding practice effects. *Mult Scler*. 2020;26(11):1298-1302. doi:10.1177/1352458519845839.

2. Rentz C, Kaiser V, Jung N, et al. Sensor-Based Gait and Balance Assessment in Healthy Adults: Analysis of Short-Term Training and Sensor Placement Effects. *Sensors (Basel, Switzerland)*. 2024;24(17). doi:10.3390/s24175598.

3. Haines TP, Russell T, Brauer SG, et al. Effectiveness of a video-based exercise programme to reduce falls and improve health-related quality of life among older adults discharged from hospital: a pilot randomized controlled trial. *Clinical Rehabilitation*. 2009;23(11):973-985. doi:10.1177/0269215509338998.

4. Brötz D, Synofzik M. Koordinative Physiotherapie für Patienten mit Ataxie.

5. Hirtz P. Koordinative Fähigkeiten im Schulsport. Berlin; 1985.

6. Boven E, Cerminara NL. Cerebellar contributions across behavioural timescales: a review from the perspective of cerebro-cerebellar interactions. *Front Syst Neurosci*. 2023;17:1211530. doi:10.3389/fnsys.2023.1211530.

7. Friedmann K. *Trainingslehre: Sporttheorie für die Schule.* 2. überarbeitete Auflage. Pfullingen: Promos; 2009. https://publishup.uni-potsdam.de/frontdoor/index/index/docid/23782.

8. Pandy MG, Andriacchi TP. Muscle and joint function in human locomotion. *Annu Rev Biomed Eng*. 2010;12:401-433. doi:10.1146/annurev-bioeng-070909-105259.

9. Chaabene H, Prieske O, Herz M, et al. Home-based exercise programmes improve physical fitness of healthy older adults: A PRISMA-compliant systematic review and meta-analysis with relevance for COVID-19. *Ageing Research Reviews*. 2021;67:101265. doi:10.1016/j.arr.2021.101265.

10. Barbuto S, Kuo S-H, Winterbottom L, et al. Home Aerobic Training for Cerebellar Degenerative Diseases: a Randomized Controlled Trial. *Cerebellum*. 2023;22(2):272-281. doi:10.1007/s12311-022-01394-4.

11. Kroenke K, Spitzer RL, Williams JB. The PHQ-9: validity of a brief depression severity measure. *Journal of General Internal Medicine*. 2001;16(9):606-613. doi:10.1046/j.1525-1497.2001.016009606.x.

12. Nasreddine ZS, Phillips NA, Bédirian V, et al. The Montreal Cognitive Assessment, MoCA: A Brief Screening Tool For Mild Cognitive Impairment. *Journal of the American Geriatrics Society*. 2005;53(4):695-699. doi:10.1111/j.1532-5415.2005.53221.x.

13. Cohen J. *Statistical power analysis for the behavioral sciences.* Second edition. New York: Lawrence Erlbaum Associates, Publishers; 1988. https://www.taylorfrancis.com/books/mono/10.4324/9780203771587/statistical-power-analysis-behavioral-sciences-jacob-cohen.
